# Context Matters: The Illusive Simplicity of Macaque V1 Receptive Fields

**DOI:** 10.1371/journal.pone.0039699

**Published:** 2012-07-03

**Authors:** Robert Haslinger, Gordon Pipa, Bruss Lima, Wolf Singer, Emery N. Brown, Sergio Neuenschwander

**Affiliations:** 1 Martinos Center for Biomedical Imaging, Massachusetts General Hospital, Charlestown, Massachusetts, United States of America; 2 Department of Brain and Cognitive Sciences, Massachusetts Institute of Technology, Cambridge, Massachusetts, United States of America; 3 Department of Neuroinformatics, University of Osnabruck, Osnabruck, Germany; 4 Max-Planck Institute for Brain Research, Department of Neurophysiology, Frankfurt am Main, Germany; 5 Frankfurt Institute for Advanced Studies, Frankfurt am Main, Germany; 6 Department of Anesthesia and Critical Care, Massachusetts General Hospital, Boston, Massachusetts, United States of America; 7 Brain Institute, Federal University of Rio Grande do Norte, Natal, Brazil; University of Southern California, United States of America

## Abstract

Even in V1, where neurons have well characterized classical receptive fields (CRFs), it has been difficult to deduce which features of natural scenes stimuli they actually respond to. Forward models based upon CRF stimuli have had limited success in predicting the response of V1 neurons to natural scenes. As natural scenes exhibit complex spatial and temporal correlations, this could be due to surround effects that modulate the sensitivity of the CRF. Here, instead of attempting a forward model, we quantify the importance of the natural scenes surround for awake macaque monkeys by modeling it non-parametrically. We also quantify the influence of two forms of trial to trial variability. The first is related to the neuron’s own spike history. The second is related to ongoing mean field population activity reflected by the local field potential (LFP). We find that the surround produces strong temporal modulations in the firing rate that can be both suppressive and facilitative. Further, the LFP is found to induce a precise timing in spikes, which tend to be temporally localized on sharp LFP transients in the gamma frequency range. Using the pseudo R^2^ as a measure of model fit, we find that during natural scene viewing the CRF dominates, accounting for 60% of the fit, but that taken collectively the surround, spike history and LFP are almost as important, accounting for 40%. However, overall only a small proportion of V1 spiking statistics could be explained (R^2^∼5%), even when the full stimulus, spike history and LFP were taken into account. This suggests that under natural scene conditions, the dominant influence on V1 neurons is not the stimulus, nor the mean field dynamics of the LFP, but the complex, incoherent dynamics of the network in which neurons are embedded.

## Introduction

Cortical processing of visual stimuli takes place in neuronal networks that are both complex and dynamic. Activity of a given V1 neuron may be influenced by thousands of synapses, only a fraction of which are directly driven by external stimuli. Most of the synaptic activity represents network interactions, both locally recurrent and long range [1]–[6]. Despite this fact, the canonical approach for understanding vision has been to ignore the network and to assume that neurons signal by increasing their discharge rate in the presence of features to which their “classical receptive fields” (CRF) are tuned. For simplified stimuli such as moving bars or gratings the receptive field model has indeed been extremely successful at explaining the spiking of V1 neurons [7]–[9]. However extending this approach towards more complex stimuli, such as natural scenes, has proven difficult [10]–[17].

Natural scenes exhibit complex spatial and temporal correlations [18]–[22], and it may be that already in V1 these correlations, mediated by long range lateral connections, and possibly also reentrant loops from higher order cortical areas, impact neuronal firing [23]–[28]. Such contextual influences would still be stimulus related, but likely not predictable by a model based on CRFs. In addition, ongoing activity generated within the network itself may influence the cells’ responses. Indeed, the spiking response of V1 neurons exhibits substantial inter-trial variability, even when identical stimuli are used [29]–[34] (but see [35], [36]). Here we quantify the relative contributions of the CRF and the surround towards the spiking of individual V1 neurons under stimulation by natural scenes movies. In addition, we analyzed the role played by both the neuron’s own spiking history and also by the global population activity reflected in the local field potential (LFP). The LFP expresses synchronous activity of local populations [37]–[39] and it has been suggested that synchronous activity plays a pivotal role in neuronal interactions [40], [41].

To disentangle the influence of CRF and surround, we presented natural scenes movies (sequences of bushes, grass and trees, and views of our laboratory) to awake macaque monkeys performing a fixation task. The movie sequences included both local motion components and also a single global motion component obtained by means of a long camera panning. We then modified the surround to generate additional movies in which the stimulus within the CRF (of the recorded neurons) was identical, but the surround differed. While the monkeys were viewing the movies, spikes and LFPs were recorded using arrays of individually controlled electrodes in V1. We fit logistic regression type Generalized Linear Models (GLMs) to the spikes, and used the maximum likelihood framework of these models to rigorously quantify the extent to which the recorded spikes were predicted by the CRF, the surround, the neuron’s own previous spiking history and the LFP.

We found that for many recorded neurons, changes in the surround resulted in different, sometimes dramatically so, stimulus locked firing. Upon inclusion of the LFP in the GLM, we further found that spikes tended to be localized on fast transients in gamma band LFPs. We used the pseudo R^2^ [42], [43] to quantify how much of V1 neurons’ spike statistics are accounted for by different influences. We found that taken collectively, the surround, previous spiking history, and ongoing LFP contributed almost as much (40%) to the total pseudo R^2^ as the CRF (60%). However, the overall values for pseudo R^2^ were small. The full (CRF plus surround) stimulus only produced an R^2^ of approximately 3% at ms precise temporal resolution, and only 5% at the temporal resolution of our stimulus (20 ms). Even when all effects, including spike history and LFP, were taken into account the 1 ms R^2^ was less than 5%. These results cast doubt upon the notion that under natural scenes conditions, V1 spiking can be understood as individual neurons driven by CRF stimuli. It is likely that visual processing in V1 is already a collective phenomenon of the population with a strong role for both laterally mediated and recurrent network effects beyond that which can be described by the LFP.

## Results

**Figure 1:**
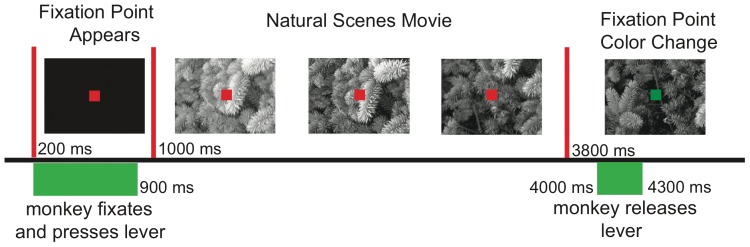
Natural scene movies with modified surrounds. Timeline for presentation of natural scenes movies. 200 ms after the start of the trial a red fixation point appeared in the middle of a black background. The monkey was required to press and hold a lever between 200 and 900 ms. The natural scenes movie (FF, AM or TR) began at 1000 ms. At 3800 ms the fixation point changed color from red to green. The monkey was then required to release the lever between 4000 and 4300 ms.

As detailed in the Methods, two macaque monkeys were trained to view natural scenes movies while fixated on a dot at the center of the screen. Each trial (shown schematically in Figure 1) started with a blank screen. At 200 ms a square red fixation point appeared in the center of the screen. The monkeys were required to press a lever within the following 700 ms and maintain their gaze. The natural scenes movie began at 1000 ms. At 3800 ms the color of the fixation point changed from red to green. To obtain a reward, the monkey had to release the lever within a window of 200 to 500 ms after the fixation point color change.

**Figure 2:**
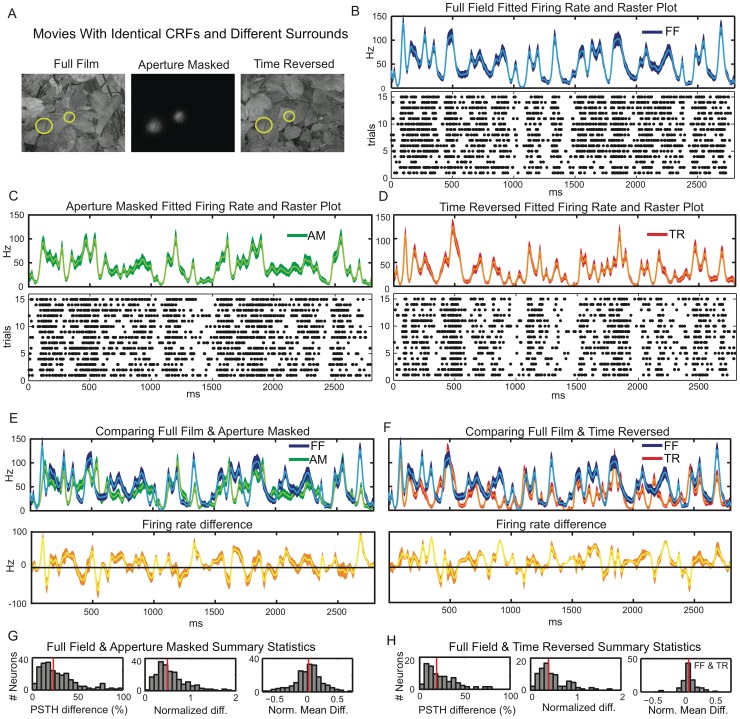
Surround context modulates response to natural scenes stimulus. **A**) Frames from one of the Full Film (FF), Aperture Masked (AM) and Time Reversed (TR) movies (see text) used to probe the influence of the stimulus surround. The movie within the CRF (yellow circle) remains unchanged across conditions, while the surrounds are all different. **B**) Raster plot and GLM fitted PSTH of a representative neuron during FF movie. The band is the 95% confidence region on the fit and the lighter line is the fit itself. **C**) Similar raster and GLM fits for the same neuron, but during the AM movie. **D**) Raster and fit for the TR movie. **E**) Top panel: Comparing GLM fitted PSTHs during FF (blue) and AM (green) movies. Bottom panel: PSTH difference (in yellow) between the FF and AM movies. **F**) Similar comparison of FF (blue) and TR (red) movies. **G**) Histograms (red lines denote medians) across the entire population of 1) the percentage of the PSTH which is statistically different between FF and AM movies 2) average of the time varying firing rate difference between FF and AM movies normalized by the mean firing rate across both conditions and 3) The difference between FF and AM mean firing rates, normalized by their collective mean. **H**) Similar histograms comparing the FF and TR movies.

To disentangle the influence of CRF and surround, each natural scenes movie was manipulated so that the portion within the recorded neurons’ CRFs remained constant, but the surround was modified. In total, three types of movies were used (Figure 2 A). The “full film” (FF) movies were unmodified. The “aperture masked” (AM) movies showed only the portion in the neuron’s CRF and obscured the remainder (surround) with an opaque Gaussian mask. Finally, the “time reversed surround” (TR) movies ran the portion of the movie outside of the CRF backwards in time. This retained the same overall illumination and contrast levels, but broke spatial and temporal correlations between the CRF and surround, with the global stimulus motion being in opposing directions. In a given experiment, all three types of movie (derived from the same unmodified movie) were presented in random interleaved fashion over multiple trials.

A standard approach towards studying natural scenes is to postulate a forward model, an explicit mapping from stimulus to spikes. However the majority of forward models have, understandably, had difficulty reproducing the trial averaged response [17]. Our goal was to quantify *all* of the stimulus related spike statistics, and a forward model would never be perfect. Therefore, we did not postulate a forward model, but took a non-parametric approach, similar to fitting a PSTH. Specifically we used a generalized linear model (GLM) in which the stimulus was included via non-parametric basis spline expansion of how the spikes depended upon the time since movie onset. The maximum likelihood framework of the GLM allows us to rigorously quantify *how much* spiking has changed as a result of surround modification. Further, it does this without postulating a detailed functional mapping, which could be suspect, of how natural scenes stimuli are translated into spikes.

In total we analyzed 305 neurons recorded with both FF and AM movies, 153 of these were also recorded during TR movies. In Figure 2B, C and D we show raster plots from a representative neuron, over repeated trials, for FF, AM and TR movies along with the GLM fitted stimulus locked firing (with 95% confidence bounds). The temporal resolution of the GLM fits is 20 ms to match the movie frame rate. Additional example neurons are shown in Figure S5. The stimulus driven spiking differs strongly (as much as 50 spikes per second) between the movies in a manner that is neither completely suppressive nor enhancing, but complex and dynamic. To more clearly show this, we plot the GLM fits for FF and AM movies on the same axes in Figure 2 E along with the difference between the two stimulus driven firing rates. Figure 2 F similarly compares the FF and TR movies. The stimulus driven firing rates are clearly statistically different over a large portion of the movie play time.

Such differences are also evident at the population level. The first panel of Figure 2 G (H) shows, for all neurons, the percentage of movie play time during which the FF and AM (TR) time varying firing rates are statistically different at the 95% confidence level (population medians of 23 and 19% respectively). In the second panel we quantify the size of the difference between PSTHs by calculating the *normalized difference between the firing probabilities* of FF and AM (TR) movies (population medians 0.49 and 0.40). This measure averages the absolute value of the difference between firing probabilities over time and normalizes by the mean firing probability. In the third panel(s) we quantify the degree to which changing from FF to AM (or TR) movies either enhanced or suppressed the mean firing rate by calculating the *normalized difference between mean firing rates* (population medians 0.02 and 0.04). This is different from the second panel because the firing probabilities are averaged to get mean rates before taking the difference. See Methods for precise definitions of these metrics.

These results are stable to the eccentricity of the CRF, see Figure S7. Moreover, these population statistics show that breaking the correlations between CRF and surround, either by surround removal (AM) or surround reversal (TR), tends not to change the mean (time averaged) firing rate from that of the original (FF) movie. However the *time varying* firing rate for the majority (but by no means all) of the neurons is strongly modulated, suggesting that the surround can play a critical role in determining the response dynamics of V1 neurons.

### Influence of Local Field Potential

**Figure 3:**
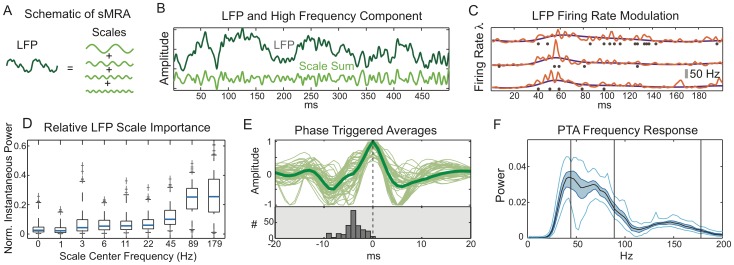
High frequency LFP oscillations impose fine timing upon spikes. **A**) Schematic of sMRA showing individually band limited scales summing to the LFP. **B**) Representative, non-sinusoidal, ongoing LFP under natural scenes stimulus and high frequency component reconstructed by summing the three highest sMRA scales. **C**) Three individual trials (identical stimulus presentations). Blue: GLM fitted spike probability without LFP included, red: with LFP included. Spikes (black dots) are preferentially located at times predicted by the GLM. **D**) The normalized instantaneous power across scales of an sMRA of the LFP dependent GLM term 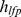 (see text) reveals the importance of each LFP scale for predicting spiking (see text). Box plots show results across the entire neural population (black lines: 50% quantiles, box edges: 25 and 75% quantiles, whiskers: 2.5 and 97.5% quantiles. Crosses denote outliers.) The scales with center frequencies of 44, 89 and 189 Hz) are most important for predicting spikes. **E**) Phase triggered averages (PTAs) of high frequency LFP. PTAs of individual neurons (thin curves) are aligned at their peaks. Thick curve is population average. Lower panel: distribution (across neurons) of preferred spiking times (relative to peak) is predominately located upon the sharp edge of the non-sinusoidal oscillation. **F**) Population averaged frequency response (black line) of PTAs is centered about 70 Hz. 50 and 95% confidence bands given by dark blue band and light blue lines.

Despite the strong influence of the CRF and surround, the stimulus is only one variable controlling the firing of V1 neuron. Refractoriness and bursting, generated by the neuron’s own biophysics, can be modeled as a renewal process [44]. We discuss this in the Methods. Another factor is trial to trial variability generated by the ongoing activity of the network within which the neuron is embedded. In principle, the spike statistics of each neuron in the network are relevant, but such information is difficult to obtain. We therefore used the ongoing LFP as a network activity surrogate. The LFP is generally assumed to reflect the *synchronous* activity of a local neuronal population [39], [45]–[47]. Since different neural processes take place at different time scales we decomposed the ongoing LFP into different frequency components (scales), which collectively sum to the original LFP, using a stationary multi-resolution analysis (sMRA) see Figure 3A and [48]. This formalism is advantageous as the dynamics in a restricted frequency range can be easily reconstructed through summing individual scales as is demonstrated in Figure 3B which shows an example reconstruction of the high frequency dynamics. Note that the high frequency LFP can be strongly non-sinusoidal with variable fundamental frequency.

We then included the ongoing LFP in the GLM as a function of both the amplitude and phase of the sMRA frequency scales (see Methods). This introduced trial to trial variability into the GLM. In Figure 3 C we show three instances (single trials) of an example neuron’s GLM fitted firing rate during a FF movie both with (red) and without (blue) the LFP scales included in the model. The ongoing LFP modulates the firing rate by the same order of magnitude as the stimulus but at a faster time scale. In other words, the LFP imposes a fine timing upon the spikes, which, although stochastic, tend to coincide with the GLM predicted elevations in firing rate.

Since the GLM constitutes a parametric model of how the spike probability depends on different frequencies and their phases, it can be used to determine which frequencies are most predictive of spiking. The functional form used to include the LFP is the GLM equivalent to the convolution of a linear filter with the LFP (see Methods). Although the frequency response of this filter can be calculated, a strong filter response at a particular frequency might merely indicate that the frequency has low power in the LFP. To determine how the spike probability is modulated by different frequencies the convolution of the LFP with the filter must be analyzed. The result is summarized in Figure 3 D. We performed an sMRA upon the LFP dependent term and calculated the mean instantaneous power of each resulting scale. This is different from performing an sMRA upon the LFP because the filter amplifies the effect of some scales and diminishes the effect of others. Although all LFP scales were included in the GLM, it is the three highest frequency scales (center frequencies of 44.5, 89 and 178 Hz) that are most predictive of the spiking. The importance of the 44.5 and 89 Hz scales agrees qualitatively with studies showing phase locking to gamma band LFP [40], [41].

The importance of the 178 Hz scale indicates a precise timing of the spikes at specific phases of sharp gamma oscillations [49] rather than a fundamental mode at that specific frequency. Fourier like decompositions of sharp oscillations involve high frequency harmonics. These harmonics represent different aspects of the same underlying oscillation and should not be considered independently. This is supported by the fact that the phases for which the GLM predicted high spike probabilities tended to be highly correlated across the high frequency scales (Figure S9). To reconstruct the underlying LFP waveform that corresponds to the strongest spiking we calculated a *phase triggered average (PTA)* of the LFP for each neuron. This is similar to a spike triggered average, but instead of triggering upon the spikes, we trigger upon the scale phases that the GLM indicates correspond to maximum spike probability. (See Methods for details.) These PTAs are shown in Figure 3 E for the entire population. The PTAs exhibit fast transients, and spiking is maximized about these transients (lower panel). The frequency response of the PTAs is centered around 70 Hz (Figure 3F). Thus although the LFP oscillation is in the gamma range, the spike probability varies at higher frequencies. The GLM uses the information in the 178 Hz scale to localize the spikes with finer temporal precision than could be accomplished without it, but the underlying LFP dynamics have a much lower fundamental frequency.

### How Predictive of Spiking is the Stimulus?

Our central goal was to quantify the proportion of spike statistics accounted for by the CRF, the surround, the spike history and the ongoing LFP. In the case of normally distributed random variables one might achieve this goal using the R^2^. This measures reduction in mean squared error. However spikes are *binary* variables and the standard R^2^ is highly inappropriate for describing them. We therefore used the pseudo R^2^ (see Methods) which is defined using the log likelihood and can be applied to binary data (see Methods and also [42], [43]). The pseudo R^2^ reduces exactly to the standard R^2^ if normally distributed variables, whose log likelihood is proportional to the mean squared error, are used.

**Figure 4:**
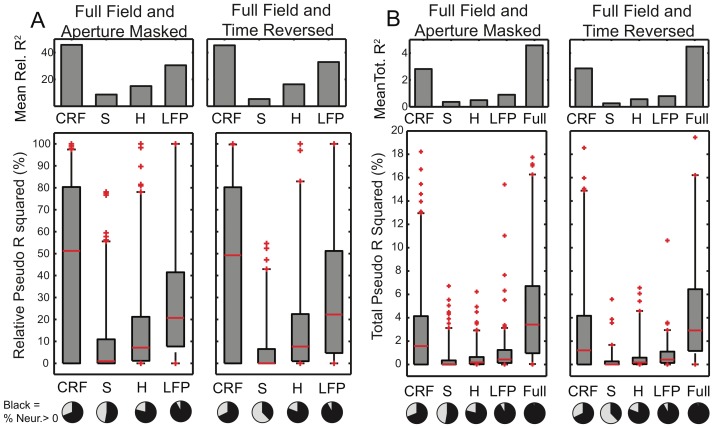
Receptive Field, surround, spike history and LFP are all important for neural spiking. **A**) Upper panels: population mean relative pseudo R^2^ (100*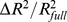) of CRF stimuli (first bar), surround (second bar), spike history (third bar) and ongoing LFP (fourth bar) for validation data. Results comparing FF and AM movies are on the left, FF and TR movies on the right. Lower panel: boxplots of distributions across all neurons. Red line = median, boxes  = 25 and 75% quantiles, whiskers = 2.5 and 97.5% quantiles. Pie charts show (in black) the percentage of neurons for which inclusion of the corresponding influence improved goodness of fit (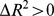). **B**) Total pseudo R^2^ accounted for by each step in the nested model, and also the full model. Although the CRF dominates, the surround, spike history and the ongoing LFP collectively account for roughly 40% of the fit. The total fit is however extremely low, 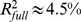
(population mean).

In Figure 4 A, we show the *relative* improvement of pseudo R of all recorded neurons for each successive addition of model complexity. That is, we normalized the successive improvements in R^2^ by the R^2^ of the full model (100*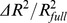), setting a scale between 0 and 100. AM results are in the left panel, TR in the right. The bar plots in the top panels give the means across all neurons. The CRF accounts for 46%, 45% (population mean, AM and TR respectively) of the fit. The surround accounts for a smaller proportion (9%, 6%), while the spike history (mean 15%, 16%) and ongoing LFP (mean 30%, 33%) account for a somewhat larger fraction.

Although the influence of the surround is much smaller than the CRF when considered across the population, there are numerous individual neurons for which it is substantial and the surround, spike history or ongoing LFP predominate either individually or collectively. The box plots in the bottom panels of Figure 4A show the distributions of relative R^2^ across all neurons. The medians of these distributions are: CRF median 51%, 49% (AM and TR respectively), surround 1%, 0%, spike history: 7%, 8% and ongoing LFP: 21%, 22%. The surround medians are near zero because not all of the neurons were responsive to the stimulus, and of those that were, somewhat fewer responded to the surround. Neurons were identified as “responsive” to an influence (CRF, surround, spike history, LFP) if inclusion of the corresponding term in the GLM improved the model’s fit to a validation data set. The percentages of responsive neurons are shown as pie charts below the box plots. 70% of the neurons responded to the stimulus. 54% responded to the surround being changed from FF to AM while 38% responded when the stimulus was changed from FF to TR. Those neurons that did respond to the surround often did so strongly. The fact that many neurons did alter their responses when the surround was changed indicates that stimulus driven spiking can not be fully explained by the properties of the CRF alone and that forward models of responses to natural scene stimuli will always be incomplete if solely based upon CRF properties.

Exactly how much of the *total* (not relative) spike statistics are accounted for by the stimulus? In Figure 4B we give the total pseudo R^2^ accounted for by the CRF (mean 2.8%, 2.9%; AM, TR respectively), surround (mean 0.4%, 0.3%), spike history (mean 0.5%, 0.6%) and LFP (0.9%, 0.8%). The distributions across all neurons are given in the lower panels. Thus under natural scenes conditions, the model including both the CRF and surround has a mean total pseudo R^2^ of 3.2% (for both AM and TR) even though the stimulus is modeled non-parametrically. It could be argued that since our stimulus has a temporal resolution of 20 ms, it is misleading to consider the statistics at the ms scale. To address this, we binned the spikes of each neuron into spike counts within 20 ms bins, and used the fitted GLM model to determine the mean firing rate within each of these bins. Then we calculated the Poisson log likelihood of each bin’s spike count, and summed over bins to determined a 20 ms resolution pseudo R^2^. This results in only a slight increase to a population mean of 5%. Note that this is the fit to the *single trial* spiking statistics. As discussed in the text S1 and Figure S6, the trial averaged PSTH is fit very well by the GLM, 92% of the explainable variance of test data can be accounted for.

**Figure 5:**
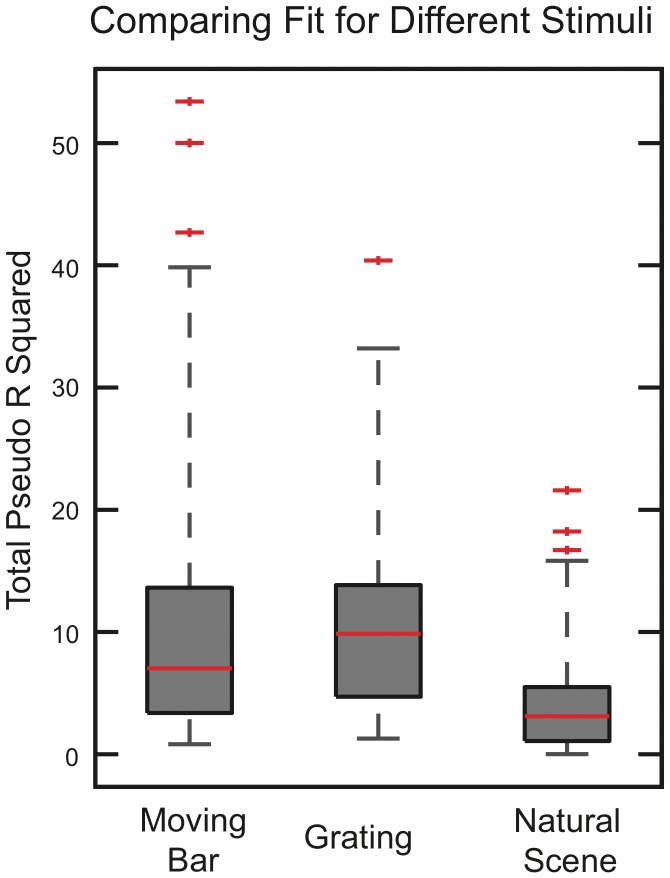
Pseudo R squared of neurons responsive to natural scenes, grating and moving bar stimuli. Box plots of total pseudo R squared accounted for by the full stimulus only (CRF + surround) for natural scenes, gratings and moving bars. The simpler “laboratory type” stimuli are better fit (median 10% gratings, 7% bars) than the natural scenes (median 3%) suggesting that the they may impose a more coherent dynamics on V1 than natural stimuli do.

It could also be argued that one should only consider neurons “responsive” to the stimulus. In Figure 5 we show the pseudo R squared accounted for by the full stimulus only (CRF and surround, but no spike history or LFP) for all neurons (80%) responding to natural scenes movies. The population mean of this distribution R^2^ = 4% (median 3%) is still low even though the stimulus is modeled non-parametrically and all neurons shown are responsive. We then wondered if the low percentage of spike statistics accounted for by the stimulus was specific to natural scenes, and if the spikes might be better explained by more “artificial” stimuli such as gratings. As shown in Figure 5, a similar calculation using grating and moving bar stimuli give mean pseudo R squareds (over the population) of 11 and 8% respectively (10% and 7% median).

For all these stimuli, the remainder of the “variance” can presumably be attributed to the detailed dynamics of the embedding network, beyond that accessible via the LFP. However the more than threefold difference in pseudo R squared between natural scenes and gratings suggests that artificial stimuli induce a much more coherent, and likely predictable, dynamics than is usually the case under natural scenes conditions. Indeed, LFP power spectra displayed a prominent gamma peak during grating stimuli that was absent during natural scenes. (See Figure S8, and also [47]). Practically modeling how neuronal spiking depends upon these dynamics requires reducing their dimensionality somehow. The LFP is a coarsely averaged mean field measure of this dynamics, and apparently an insufficient description. Exactly which reduced representation would be sufficient is currently unclear, although an obvious place to start would be to include the spiking of simultaneously recorded cells.

## Discussion

Investigations of the neural code have historically focused upon how single neurons respond to stimuli [29]. In many systems, this approach has led to an observation that many neurons have “preferred” stimuli, corresponding to an increased discharge rate. Indeed the concept of the classical (and other types of) receptive field has guided investigations of V1 since Hubel and Wiesel introduced it [7]. However most V1 neurons are strongly variable trial to trial, even when identical stimuli are presented [30]–[34]. In addition, the majority of V1 studies have employed simplified “laboratory” type stimuli such as gratings or moving bars (see [50] for a review). These issues raise the question of how dominant CRFs are when more “naturalistic” stimuli are used, or if the surround and other factors have increased importance. In this paper we undertook to determine exactly how much of V1 neurons’ spiking (quantified by the pseudo R^2^) is due to the CRF versus the surround when naturalistic stimuli are used. We also quantified the roles played by the neuron’s own spike history dependent biophysics, and by the average population activity (LFP).

We found that not only did all of these factors modulate the spiking probability of V1 neurons, but that taken collectively the surround, spike history and LFP were of nearly equal importance to the CRF (60 versus 40%). We note that for each natural scene movie, we only employed two surround modulations, aperture masked and time reversed. Had more modulations been used, the influence of the surround might have been stronger compared to the CRF, although this would depend upon the modulations’ exact nature (naturalistic versus white noise for example). Regardless, the entire stimulus (CRF and surround together) explained a relatively small percentage of the spike statistics, R^2^ = 3% (population mean) at 1 ms resolution, and R^2^ = 5% at 20 ms resolution. For natural scenes, the CRF, and indeed the stimulus as a whole, is only the tip of the iceberg.

Our study showed strong surround modulation of the V1 neuronal response to natural stimuli. This modulation was both facilitative and suppressive, often in the same neuron. Indeed, the mean firing rates of many neurons varied little. Of particular interest is that modulation was observed not only between FF and AM movies but also between FF and TR movies. Thus the dynamic modulation we observe is evidence of a complex non-linear interaction between CRF and surround, not merely a function of the surround’s presence or absence. Several prior studies have varied the size of a natural scenes stimulus [10], [11], [51], [52]. However to our knowledge ours is the first that has changed the correlational (between center and surround) structure of naturalistic stimuli and demonstrated a similar dynamic modulation. We note that this has been done for artificial stimuli, see for example [53].

It should be noted that determining the exact boundary of the receptive field is difficult, and can depend the exact method used to map it. Barlow used small moving bars and edges to determine the spatial extent of the excitatory region, or “minimum response field” (MRF) [54]. Reverse correlation methods using either bars [55] white noise [56], randomly flashed squares [10] or other artificial stimuli are also commonly used. A third technique is to increase the size of a grating patch and denote the RF as the patch size for which the response no longer increases [57], [58]. This third technique tends to give larger estimates than the MRF or reverse correlation techniques, and has been noted to depend upon grating contrast [25]. Other researchers have modeled both the excitatory center and inhibitory surround using Gaussian based models [59]. Good discussions of these issues can be found in [25], [59], [60]. In our case, we used a reverse correlation type procedure that employed a long moving bar stimulus and the computation of an average multi-unit activity response. Since we used MUA it is possible that for some neurons our apertures contained some of the proximal inhibitory surround. However it should also be noted that some studies have suggested the size of the distal surround to be up to five times that of the CRF [26].

Surround suppression of the V1 neuronal response has long been noted by studies using grating type stimuli in anesthetized cats and monkeys [28], [59]–[62] and also during the free viewing of natural scenes by awake monkeys [51]. Others have found surround driven changes in grating orientation tuning [63], [64]. Occasionally surround facilitation has been noted, but deemed weak [65]. A recent grating based study in anesthetized cats has placed the number of V1 neurons exhibiting surround facilitation at 6% [66]. This result is at odds with our study in which many neurons displayed both facilitation and suppression. The difference may lie in the use of natural scenes versus gratings. Indeed, it has been noted by others that contrast levels can dictate whether the surround is suppressive or facilitative [67].

Another possibility is that surround suppression versus facilitation is a function of distinct neuron types. Haider et. al. [52] performed intracellular recordings in anesthetized cats while varying the size of a natural scenes stimulus. They found that excitatory, regular spiking pyramids tended to exhibit surround suppression, while fast spiking interneurons exhibited enhancement. This study also found that regular spiking neurons tended to spike more reliably when the surround was included, and hypothesized that increased activity of inhibitory cells enforced a sparser code in the pyramids. Several earlier studies have speculated on the role of a natural scenes surround in enforcing sparsity, demonstrating not only sparser coding [11], but also improved information transmission by neurons [10]. These studies, and our own strongly indicate that interactions between CRF and surround are fundamental for sensory processing in V1.

In spite of this, forward modeling studies of how visual stimuli are transformed into spiking have tended to focus on estimations of neuron’s spatio-temporal receptive fields (STRFs). Sometimes these are coupled with a non-linearity (LN Model) [56], [68] and/or a Poisson spike generator (LNP model) [69]. This approach has achieved some success in predicting responses to grating orientation and tuning, although contrast induced non-linearities have often been noted, (see [50] for a review). STRF type models have also been used to capture the response of V1 simple cells to natural scenes [12], [17], [68], [70] More complex models, such as spike triggered covariance (STC) [15], [71] have also been used to describe the response to natural images, particularly the response of complex cells. Forward model quality has generally been evaluated by comparing predictions to the PSTHs of repeated trials in a validation set. When corrected for finite data sizes, this is the “percentage of explainable variance” of Gallant and colleagues. For natural scenes, the percentage of variance explained in V1 has tended to be no more than 40% [17] These results should be contrasted with studies in the LGN [72] which have achieved much better (∼80%) predictability). It should be noted that the explained variance is a very different measure than the pseudo R^2^ (see below). Possible reasons for poor performance in V1 are temporal non-linearities, center surround interactions, and complex network activity, none of which are captured by the STRF.

Our goal was different from that of forward modelers. We wanted to quantify, on an absolute scale, how much the spiking was driven by the CRF versus the surround. For this reason we employed the pseudo R^2^ [42], [43] to quantify the single trial statistics, rather than the explained variance. We also did not attempt a forward model, but instead used a non-parametric, basis spline based, model for the stimulus. This has the advantage that the explained variance is extremely high (96% for training and 92% for test data, see Figure S6) and one does not have to worry that the stimulus model is “wrong” when trying to quantify how important different effects are on the spikes. Our results show that the CRF is not sufficient, even for describing the trial averaged response, suggesting that forward models employing a STRF type filter, localized within the CRF, will always be problematic for natural scenes stimuli and that center surround interactions must be included. Further, the trial averaged response is an exceedingly poor descriptor of the single trial statistics, suggesting that if vision is to be understood, the stimulus must be treated as a small perturbation of an individual neuron’s dynamics.

Spikes are binary variables with a ms precise, time scale dictated by their width. It has been shown that neurons can respond with ms precision to sharp changes in membrane potential [73] and are capable of learning fine timing encoding representations [74]. It is therefore important to use a model that operates at fine temporal resolution and also respects the binary character of the data. Our spline-based stimulus model was nested in a GLM which simultaneously also models the influence of the spike history and LFP. The dynamics of the stimulus, spike history and LFP all have different time scales. The GLM provides a multiscale model of how they influence the neuron at the time scale appropriate for describing spikes. Still, an argument that could be put forward is that since the stimulus changes at a slower (here 20 ms frame rate) speed, any analysis of its importance should be based upon this time scale. At a 20 ms time scale, the data is described by spike counts, Poisson variables from which a pseudo R^2^ can also be calculated. When we did this, we found that the natural scene spike counts were slightly better fit than the individual spikes, but not dramatically so. Thus even at stimulus’s own time scale, neuronal activity is not well described. We note that the situation may be different for experiments that generate more coherent neuronal activity, such as those that use grating type stimuli or anesthetized protocols. Indeed we found we could fit the single trial spiking statistics of both grating and moving bar stimuli with much higher accuracy than natural scenes movies (Figure 5).

Most likely, the dominant factor driving V1 neurons is the network, a view supported by the fact that recurrent, lateral and top down connections dominate over feed forward [25], [75], [76] and the fact that ongoing network states are known to strongly influence spiking [77], [78]. In the absence of detailed information about the network, we used the LFP as a surrogate. It is important to recognize that the LFP is a population averaged measure of local network activity, whose exact meaning is strongly debated. We found that spikes tended to be localized on sharp LFP transients in the gamma range. This indicates that during natural scenes viewing the ongoing LFP carries information important for predicting spike timing despite there being no gamma peak in the LFP power spectrum. (Figure S8) It was the use of the PTA, based upon the nested GLM, that allowed us to uncover this feature.

Many studies use the spike triggered average (STA) or spike triggered spectrogram to make inferences about how spikes depend upon the LFP [47], [79], [80]. These measures average the LFP (or its power spectra) as a function of when spikes occur. In contrast, the PTA leverages the GLM spike probability model to average the LFP when spikes are most *probable*. These are not the same, because spikes do not always occur when they are maximally probable. Directly comparing the PTA and the STA (see Methods below) shows that the STA largely reflects a deflection *after* the spike. In contrast the PTA reveals the entire feature (sharp transient) that increases the spike probability. Another difference between the approaches is that our nested GLM takes into account the effect of stimuli and previous spiking history in addition to the LFP. Thus it can dissociate between the case when spiking and LFP are being simultaneously driven by the stimulus, and are thus correlated, and when spikes are correlated with the LFP independent of the stimulus.

The observation that spike timing is coupled to LFP oscillations is not new. However the majority of studies comparing LFPs to spikes have focused on either spectral power, or phase relationships between low (<10 Hz) frequency LFPs and multiunit activity (MUA) [47], [81]–[83]. In contrast we found phase relationships between the gamma band and individual spikes. At these higher frequencies, it has been shown that increased gamma power correlates with MUA [38], [47], [84]. Further, intracellular studies have shown that inhibitory neurons, thought to be involved in gamma, tend to fire in the gamma trough [85]. Still, studies of phase relationships have tended to be confined to grating stimuli, anesthetized animals, or both [38], [86]–[88]. Based upon these and similar studies, it has been hypothesized that gamma implements a temporal coding scheme (see [46] for a review). However gamma power has been shown to be a function of grating contrast [89] and it is also known that the LFP power spectrum is sharply modulated by different (grating versus natural scene) stimuli [90] (and see Figure S8). Our results suggest that even when gamma is incoherent, as during our natural scenes stimulus, it may still induce timing codes and play a computational role.

LFPs provide one measure of network activity, and indeed the pseudo R^2^ of the LFP portion of the GLM was comparable in magnitude to that of the stimulus (Figure 4A). However, the overall fit, even including the LFP was poor (R^2^<5%). This suggests that under natural scenes conditions, the dynamics of the V1 network are highly complex, and neither the stimulus, nor the LFP, are the dominant drivers of V1 neurons. Instead, ongoing and mostly incoherent network activity driven by input from other cortical areas or even processes intrinsic to V1 predominates at the single neuron level. At some point in the visual pathway, information must be combined into a collective representation. Our results suggest that this is already happening in V1 and that vision must be considered as a tightly integrated, and complex, phenomenon of the network, not the sum of the individual neurons receptive fields.

## Methods

### Training and Visual Paradigm

Two rhesus monkeys were used in this study. All experimental procedures were approved by local German authorities (Regierungspraesidium, Hessen, Darmstadt) and were in accord with the guidelines of the European Community for the care and use of laboratory animals (European Union directive 86/609/EEC). In order to ameliorate suffering and improve the well being of the monkeys, we employed the following practices and techniques. The monkeys were housed in groups of 3 to 5 animals within large spaces and with access to open air. During training, the monkeys were taught to spontaneously come to the primate chair without need of restraining collars. Titanium head-fixation implants and recording chambers were fixed directed to the bone without the use of acrylic cement. These techniques are less invasive and contributed to the monkeys’ quality of life. A camera based non-invasive technique was used for monitoring eye movements, precluding the use of scleral search eye coils. Finally, the recording sessions were always interleaved with long recovery periods.

A detailed description of the training paradigm and recording procedures is given elsewhere [91]. Briefly, each trial started with a blank screen and then, at 200 ms, the appearance of a 0.15° square red fixation point (4×4 pixels; luminance, 10.0 cd/m^2^) centered in the screen. The monkeys were required to press and hold a lever within the following 700 ms, and to maintain their gaze within a ∼1°×1° window. At 3400 ms the color of the fixation point changed from red to green. To obtain a reward, the monkey had to release the lever within a window of 200 to 500 ms after the fixation point color change. Trials were aborted whenever early or late lever releases occurred, or whenever fixation was interrupted. Eye position was monitored by an infrared eye tracker (Matsuda et al., 2000; temporal resolution of 33 ms). See Figure 1 for a schematic timeline of the experiment.

### Visual Stimuli

Test stimuli consisted of natural scene movies recorded with a digital video camera (resolution 960×720 pixels at 30 frames per second, non-interleaved, Panasonic DVCPRO-HD format). All video clips were fully desaturated and converted into bitmap image sequences cropped to a size of 936×702 pixels. The sequences were displayed at 100 Hz (the same frame was presented twice) using a standard graphical board (GeForce® 6600-series, NVIDIA®, Santa Clara, USA) controlled by ActiveStim (www.activestim.com) (average luminance, 10 cd/m^2^). This software allowed high timing accuracy and stimulus onset jitters below one millisecond. The cathode-ray tube monitor used for presentation (CM813ET, Hitachi, Japan) subtended a visual angle of 36°×28° (1024×768 pixels).

A total of 7 different video sequences were used in this study. They consisted of images of leaves, garden trees, or scenes in our laboratory obtained after a single panning movement of the camera (i.e., the video sequences always contained a single predominant global movement component). The movies (irrelevant for the task) were always presented 800 ms after fixation onset and lasted 2800 ms until the fixation point color change. In a given experiment 300 presentations of a single video sequence were made to the monkey. These trials were split equally into 3 stimulus conditions, generated from the same video sequence. In Condition 1 the full frame (FF) was presented. In Condition 2 (Aperture Masked, (AM)), only the portion of the film within the CRF was presented. The remainder was obscured by an opaque Gaussian mask that prevented any sharp edges in the image. Finally, in condition 3, the surround (visual field external to the CRF) was presented reversed in time while the region within the aperture remained unchanged (TR condition). The CRF and surround were blended using Gaussian masks so that no sharp edges were generated in the images. Example frames of these stimuli can be seen in Figure 2A. and also see Figure S1.

We also recorded moving bar (see below) and grating stimuli for comparison with the natural scenes movies. Grating stimuli had spatial frequency ranging from 1.25 to 2.0 cycles per degree and velocity ranging from 1.0 to 1.5 degrees/s orthogonal to their orientation). These values were chosen because they elicited robust average responses in V1. The gratings were square wave functions and had a duty cycle of 0.3. Moving bar stimuli were the same as used for mapping the CRF and are described below.

### Aperture Mask Generation

Apertures were created online, individually for each recording electrode as follows. At the beginning of each recording session, CRFs were mapped using an automatic procedure in which a bar (1000×5 pixels, corresponding to 39×0.2° in visual angle) was moved across the screen in 16 different directions (N = 160 trials). CRF maps were obtained by computing an average matrix, in which the responses were added in 10 ms bins (corresponding to 0.2° in visual angle) for all directions. This method allowed us to estimate precisely the center and size of the aggregate CRFs (MUA) for a given electrode. Based on these parameters we were able to determine the position and the width of the aperture mask. For most of the experiments we selected the best electrode for unit isolation and responsiveness. A single aperture mask was used in these cases. Occasionally, two aperture masks were used for non-overlapping CRFs located, respectively, at the central (2 to 5 degrees of eccentricity) and peripheral (10 to 14 degrees) representations of the visual field.

**Figure 6:**
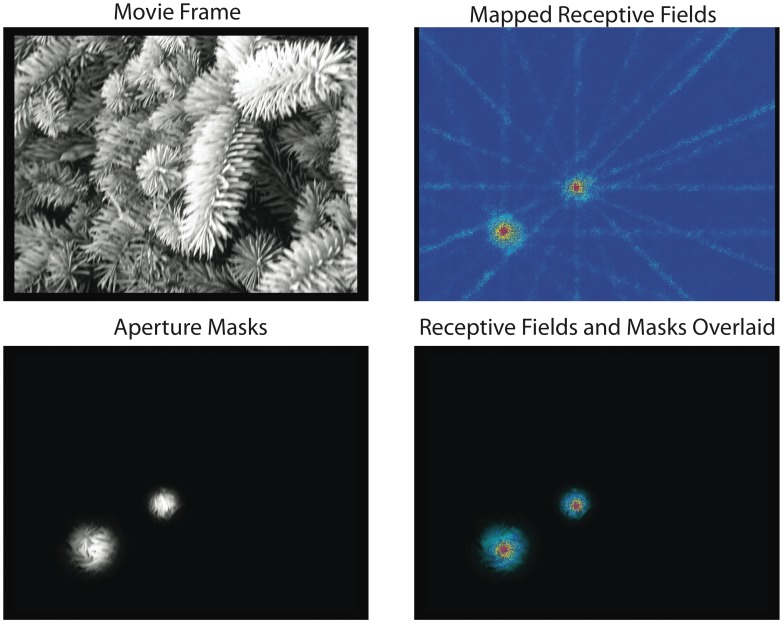
Three examples of aperture mask placement. Top left: movie frame. Top right: CRFs of multiunit activity from two recording electrodes. Lines are artifacts resulting from the use of bars moving in discrete directions for CRF mapping. Bottom left: aperture masks generated on-line. Bottom right: aperture masks overlaid on CRFs. Note that masks fully contain CRFs.

Each aperture mask was adjusted as function of CRF size in a way that the CRF‘s hot spot was always fully covered by the mask (see Figure 6 for an example, and Figure S1 for additional examples). This procedure was performed visually based on the obtained RF maps. We used several different sizes of apertures, (30,40,50,60,70 pixels corresponding to 1.17, 1.57, 1.96, 2.35, 2.74 degrees of visual angle). Our aim was to ensure that our apertures contained the full CRF. Thus we erred on the side of caution and it is possible that our apertures contained part of the proximal surround. As an additional check however, we varied the aperture size for a subset of the experiments and found the results to be relatively stable in the 30–70 pixel range (see Figure S2).

### Recording Procedures and Data Collection

Recordings were made from the opercular region of V1 (receptive fields centers, 2° to 5° eccentricity) and from the superior bank of the calcarine sulcus (10° to 14° eccentricity). Electrodes were inserted independently into the cortex via guide tubes positioned above the dura (diameter, 300 µm; Ehrhardt Söhne, Germany), assembled in a customized recording device (designed by one of the authors, SN). Quartz-insulated tungsten-platinum electrodes (Thomas Recording, Germany; diameter, 80 µm) with impedances ranging from 0.3 to 1.0 MΩ were used to record simultaneously the extracellular activity from 4 to 5 sites in both superficial and deep layers of the cortex.

Spiking activity of small groups of neurons (MUA) and the local field potential (LFP) were obtained by amplifying (1000X) and band-pass filtering (MUA, 0.7 to 6.0 kHz; LFP, 0.7 to 170 Hz) the recorded signals with a customized 32 channels Plexon pre-amplifier connected to an HST16o25 headset (Plexon Inc., USA). Additional 10X signal amplification was done by on-board amplifiers (E-series acquisition boards, National Instruments, USA). The signals were digitized and stored using a LabVIEW-based acquisition system developed in our laboratory (SPASS, written by SN. Spike sorting and identification is discussed in detail in the Text S1 and see Figure S3. The LFP was acquired with a temporal resolution of 1 ms. We performed careful controls to determine the extent to which the spikes were “leaking” into the LFP and concluded that the effect of leakage upon our GLM fits was small compared to the influence of other features in the LFP. These controls are detailed in Figure S4 and the Text S1.

### Nested Logistic Regression Models

Quantifying the relative contributions of different influences upon a neuron’s spiking statistics requires that they be considered within a single unified modeling framework. Spikes are stereotyped binary events localized in time. Logistic regression, a type of Generalized Linear Model (GLM), allows for the simultaneous regression of multiple influences on binary data at fine (here ms) temporal resolution. To quantify the role played by different influences, such models can be *nested*, that is made progressively more complex by sequentially adding the effects of the CRF, surround, spike history and LFP to the model.
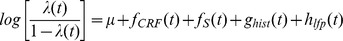




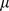
Is a parameter denoting a mean firing rate. 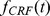 models the stimulus within the CRF and 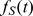 that of the surround. 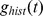
and 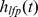 model the previous spiking history, and LFP respectively. The above model was partitioned into a series of models of increasing complexity, or nested models.

_1)_ Mean Firing Rate Model: Poisson spiking is assumed. This model has one free parameter (the mean firing rate) and is the null model.
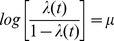



2) CRF Model: It is assumed that only the stimulus in the classical receptive field is important. We do not attempt to create a forward model of the how the stimulus generates the spikes. Instead we take a non-parametric approach and use a linear combination of 4th order B-spline basis functions generated using a knot spacing of 20 ms chosen to match the movie frame rate. Since the CRF does not change between the FF, AM, and TR movies, all three are modeled by the same basis spline expansion which subsumes both the mean firing rate and any CRF induced time varying modulation of this firing rate.
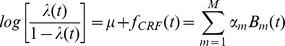



The basis functions 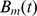
are functions of the time since the beginning of the movies, and the 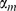
are parameters fit by logistic regression. The result of this approach is essentially a smoothed PSTH, and had a first order B-spline basis been used the model would be identical to a PSTH. Such splines provide extremely accurate smooth fits, see Figure S6. Although our non-parametric approach does not tell us *how* the stimulus is translated into the spikes, it allows us to *quantify* the stimulus’s influence, more specifically that of changes in the surround, upon the spikes.

3) CRF and Surround Model: It is assumed that the surround is important, and therefore the movies (FF, AM and TR) are each modeled by a separate basis spline expansion.
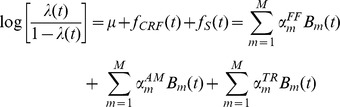



**Figure 7:**
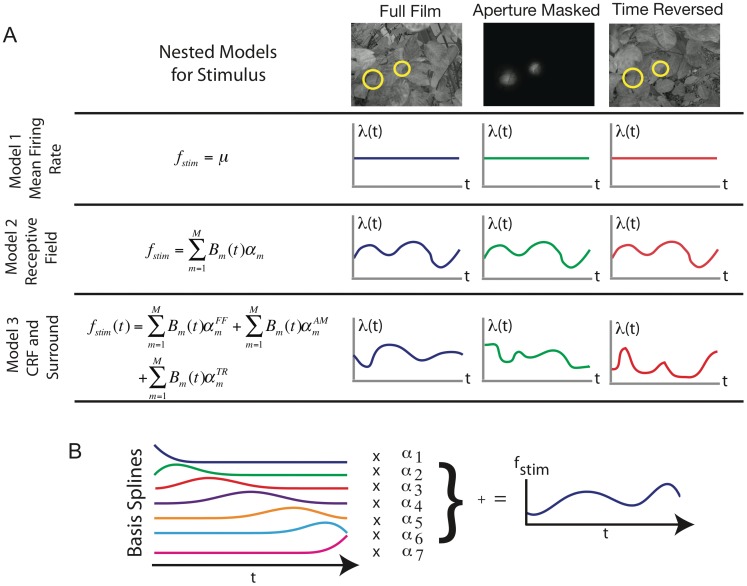
Equations and schematic representation of the three nested models for the stimulus 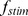. A) Model 1: Mean firing rate model assumes Poisson spiking for all three (FF, AM, TR) conditions. Model 2: Receptive field model assume modulation by the portion of the stimulus within the CRF. All three conditions are modeled non-parametrically with the same basis spline expansion because the CRF is identical in all three conditions. Model 3: CRF and surround model. The surround changes between the conditions and therefore each is modeled with a separate basis spline expansion. B) Schematic of the basis spline expansion. A linear combination of 4-th order basis splines, functions of the time since the onset of the natural scenes movie, was used to model the effect of the stimulus. The parameters 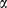 are fit in the logistic regression model.

In effect, this makes separate smoothed PSTHs for the FF, AM and TR movies. See Figure 7 for a graphical explanation of the stimulus terms.

4) Receptive Field, Context and Spike History Model. The effects of the previous spiking history are added.
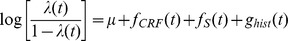



The influence of the previous spiking history is modeled as a function of the time since the most recent spike. Again a linear combination of B-splines is used.
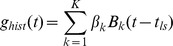



**Figure 8:**
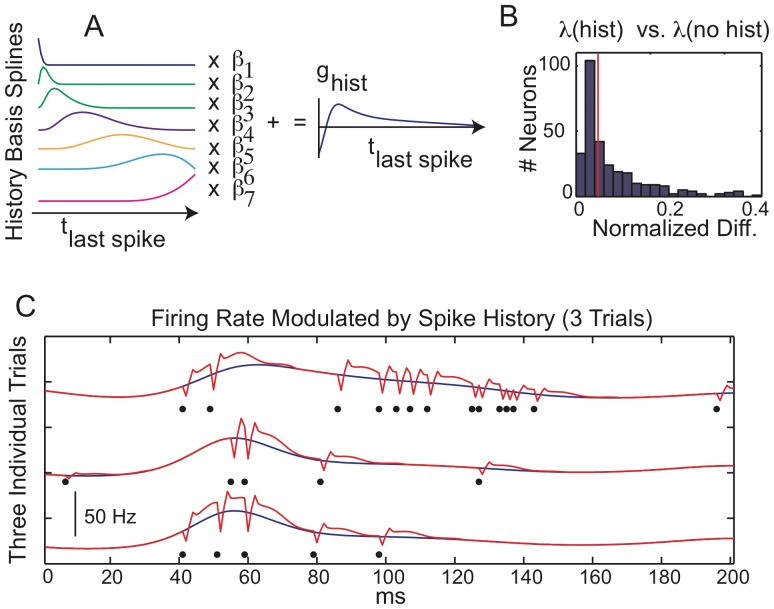
Modeling the effect of previous spiking history. A) Schematic of basis spline expansion for the spike history dependent term 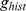 of the logistic regression model is a function of the time since the most recent spike. B) Histogram across all neurons of the difference in the firing probabilities with and without history normalized by the mean firing rate. C) Fitted spike probability for three identical stimulus presentations without (blue) and with (red) the spike history term included in the model. Black dots indicate the spikes.

The 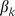
are parameters fit via logistic regression and 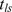 is the time of the most recent spike. We used 8 knots spaced logarithmically at [0,1,2,3,5,9,15,25] ms. By restricting the effect of the previous spiking history to 25 ms, we avoid interactions between the history and stimulus terms. This model form can capture both refractoriness and bursting. See Figure 8 for a graphical explanation of the history term and examples of how it modulates the spike probability.

5) Receptive field, Context, Spike History and trial varying LFP model. Trial to trial variability (as reflected by the LFP) is included.
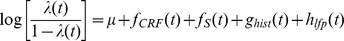



The functional form of 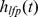 is described below.

**Figure 9:**
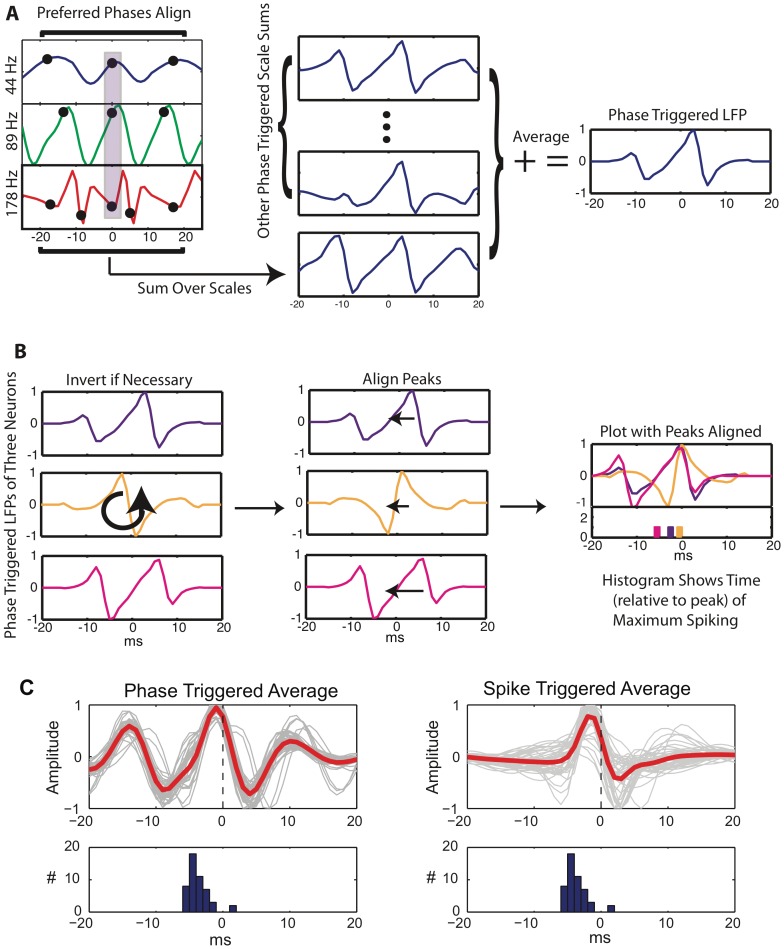
Phase triggered averaging. A) Schematic of phase triggered averaging (PTA) for a single neuron. Times for which the three high frequency scales have their preferred phases are identified, and the sum of the three scales about these times is averaged over all instances. B) PTAs are compared across neurons by inverting them (if necessary) so that all have increasing derivatives at t = 0. The peaks are then aligned, and all PTAs plotted together with a histogram showing the time (relative to the peak) at which the spike probability is maximized. C) Comparison of PTA and spike triggered average (STA) for using only the three high frequency scales. See text for discussion.

### Including Time Varying LFP in the Model

To obtain a trial specific measure of the population activity we subtracted the evoked (trial averaged) LFP from the ongoing LFP. We then decomposed it into band limited LFP time “scales” 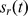 using a stationary multi-resolution analysis. This preserves the dynamics of the original signal in that the scales collectively sum to the original LFP.
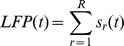



The individual scales 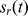
are band limited, with center frequencies that scale as powers of 2. Summing a subset of the scales immediately reconstructs the dynamics in a restricted frequency range. It is important that a *stationary* MRA be performed so that the decomposition is shift invariant. A standard discrete wavelet transform based decomposition will not be shift invariant because of the discrete tiling of the time frequency plane *(1)*. The Matlab function swt.m can be used as the basis of the sMRA.

Once the scales were found, they were separated into stimulus locked and trial to trial varying LFP scale components.
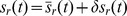
where 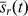
is the scale averaged over trials with identical stimulus presentations (FF, AM or TR) and 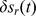
is the trial varying component. We only use the trial varying component because any trial averaged component is implicitly included in the stimulus locked basis spline expansion.

We calculated the instantaneous amplitude and phase of each scale’s trial varying component. The imaginary part 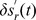 was found using a Hilbert transform (MATLAB hilbert.m function and the instantaneous amplitude and phase of each scale calculated as.
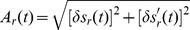
and



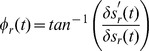



We included the amplitude and phase in the logistic regression by assuming that the dependence was oscillatory and scaled with the amplitude of the oscillation. E.g.
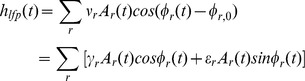



The second equality follows via a trigonometric identity, and allows the LFP to be included in the logistic regression model as a sum of terms linear in the parameters. Inclusion of phase term allows the LFP dynamics to be captured and inclusion of the amplitude allows the overall magnitude of the dynamics to be captured. We tested model forms with higher order terms in the phase and found no improvement in model fit. Models that did not include the phase, but just the power (amplitude squared) of the scales, provided no improvement in fit over models that did not include the LFP at all.

### Model Validation

For each neuron, the set of experimentally recorded stimulus trials was split into training (70% for model fitting) and test (30%, for model validation) data. We used the log likelihood of the validation data to test whether each step in the nested model was justified. In the case of normally distributed random variables the log likelihood is proportional to the residual sum of squares. Thus improvements in the log likelihood have both an intuitive and statistically rigorous, connection to error reduction. The log likelihood for binary data can be written as.
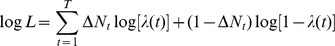
Where 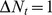 if there is a spike in bin t and is 0 otherwise, and the sum is over all time bins. We also calculated the changes in the Akaike and Bayesian Information Criteria and used a discrete time Kolmogorov Smirnov test [92], [93]. These results are displayed in Table S1, along with the percentages of neurons that passed the log likelihood validation procedure described above.

### PSTH Difference Measures

To quantify the differences between two GLM fitted PSTHs we used three different statistics. Notation below assumes FF and AM fits are being compared, but identical statistics are used to compare FF and TR fits.

1) The *percentage of time* that the two PSTHs were statistically different. This was determined from 95% confidence bounds on the GLM fits. This procedure is explained in detail in the text S1.

2) To quantify the size of the difference between PSTHs we calculated the *normalized difference between firing probabilities.* This averages the absolute value of the difference between firing probabilities over time and normalizes by the mean firing probability.
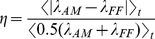



3) To quantify the degree to which changing from FF to AM (or TR) movies either enhanced or suppressed the mean firing rate we calculated the *normalized difference between mean firing rates.* This is different from 2) because the firing probabilities are averaged to get mean rates before taking the difference.
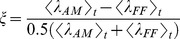



### Pseudo R Squared

To quantify overall goodness of fit we used the pseudo R^2^.
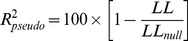



[42], [43] 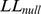
is the log likelihood of the mean firing rate (null) model, for which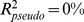. If instead the spiking is described exactly, 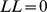, and 
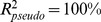
. For Gaussian random variables, the log likelihood is proportional to the variance and the pseudo R^2^ reduces to the commonly used R^2^. Use of this measure can also be thought of as performing a deviance type goodness of fit analysis [94], [95]. To compare the importance (improvement in fit) resulting from different terms in the logistic regression model at 1 ms temporal resolution, we used ratios of the increase in pseudo R^2^ after inclusion of the term to the pseudo R^2^ of the most complicated of our fitted models (model 5).

To calculate pseudo R^2^ at 20 ms resolution, we binned our spikes into spike counts 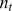
within nonoverlapping 20 ms bins. We then averaged the spike probability over each 20 ms bin to get a mean firing rate r within the bin. Finally we calculated the Poisson log likelihood of each bin’s spike count and used this to construct the 20 ms resolution pseudo R^2^. That is, 
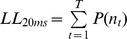
 where the sum is over 20 ms bins and 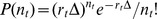 is the Poisson probability of a firing rate 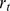 producing spikes within a bin of width 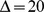
ms.

### Phase Triggered Averaging

An sMRA of 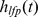
and subsequent calculation of the mean instantaneous power of each resulting scales, indicated that the three highest frequency scales were most predictive of spiking. These three scales were used to calculate *phase triggered averages* (PTAs) of the LFP. This is similar to calculating a spike triggered average (STA) but instead of triggering the average upon spikes, the triggering is upon the LFP scale phases which correspond to the highest probability of spiking in the logistic regression model. The procedure is described schematically in Figure 9. In brief, the PTA of a single neuron is calculated from the three high frequency scales by first locating all instances where these scales have their “preferred” phases. In a [−25 25] ms epoch surrounding these instances the sum of the high frequency scales is then averaged analogously to calculating an STA, but triggered on the phases instead of on spikes (Figure 9 A). To compare these PTAs across neurons we used the procedure presented schematically in Figure 9 B. First we inverted all PTAs with negative derivatives at t = 0 so that all PTAs had positive derivatives. Second we shifted all PTAs so that their peaks aligned. Finally we made a histogram of the time of maximum spike probability, relative to the PTA peak.

The PTA is similar to an STA but the STA is a measure computed directly on the data (averaging of the LFP based on when spikes occur) and the PTA is a measure computed from a fitted model (averaging the LFP based upon fitted model parameters). Thus the PTA reflects what the LFP (or frequency restricted LFP) looks like when spikes are most *probable*. The STA reflects what the average LFP is when spikes *occur.* We compare the two in Figure 9C.

## Supporting Information

Text S1 Details on spike sorting, control of LFP “leakage” into spikes, calculation of confidence bounds on GLM fits, goodness of fit of GLM to trial averaged PSTHs and the effect of eccentricity on results.
(DOC)

Figure S1 **Three examples (columns) of aperture mask placement.** Top row: movie frame. Second row: CRFs of multiunit activity of recording electrodes. (Example in left column records from both 2-5 degrees eccentricity and 10-14 degrees, i.e. two different electrodes). Third row: aperture masks generated on-line. Bottom row: aperture masks overlaid on CRFs. Note that masks fully contain CRFs.
(TIF)

Figure S2 **Varying aperture mask size.** The percentage of the PSTH that was statistically different (at 95% confidence levels) between FF and AM movies (upper panel) and the normalized difference in time varying firing rates (lower panel) for different sized apertures. There are (38, 13 38, 38, 38, 19) neurons for the (30, 50, 70, 100, 150, 200 pixel) diameter apertures respectively. Corresponding diameters in degrees of visual angle are given in the figure.
(TIF)

Figure S3 **Spike waveforms isolated from multiunit activity.** Waveforms of the three neurons whose PSTHs are presented in the paper (Figure 1 & Figure S4) are shown in red. Grey shows non-isolated background spikes (MUA).
(TIF)

Figure S4 **Quantifying spike leakage into the LFP.** A) Spike triggered averages from 9 representative neurons. Dark blue: STA of original LFP, Light blue: STA of compound LFP generated using original spike times, Red: STA of compound LFP generated using altered spike times. STAs of the original and first compound LFPs are highly similar indicating that our procedure for generating compound LFPs works properly. The STA of the second (red) compound LFP is, in contrast negligible with only minor leakage effects. B) Histogram of log likelihood increase, upon inclusion of LFP in a GLM model, of the second (altered spike time) compound data normalized by the log likelihood of the first (original spike time) compound data. For 70% (out of 44 neurons shown) the control (altered spike time) data has a log likelihood increase less than one fifth (20%) that of the original data. C) Scatter plot of the LFP induced increase in log likelihood for the original and control data. Each dot represents a single neuron. Red line is a linear regression.
(TIF)

Figure S5 **PSTHs of two additional V1 neurons.** These exhibit significantly different stimulus locked firing responses to natural scenes stimuli when the surround is changed but the CRF stimulus is not. As in the main text, upper panels show GLM fitted “PSTHs” (blue  =  FF, green  =  AM, red  =  TR) and lower panels show differences (in yellow) between PSTHs. Lighter lines are the PSTHs and differences while the dark bands denote 95% confidence regions.
(TIF)

Figure S6 **Spline based GLM models accurately fit trial averaged firing rate (PSTH).** A) PSTHs (20 ms histogram) and spline fits (red) for two example neurons under natural scenes stimulation. B) Distribution of explained variance of training data (left), test data (middle) and test data corrected for finite number of test data trials (right). C) Splines used to non-parametrically model the stimulus drive tiled the entire 2800 ms span. Here we show a subset. Colors are visual aid to distinguish adjacent splines.
(TIF)

Figure S7 **Comparing differences between the PSTHs as a function of eccentricity (2-5 degrees versus 10-14 degrees).** A) Percentage of PSTH statistically different, B) normalized difference between PSTHs, C) normalized mean firing rate difference between PSTHs. Distributions are all identical (via KS test) between 2-5 and 10-14 degrees except for the normalized mean firing rate difference between FF and TR (p = 0.049).
(TIF)

Figure S8 **Grating stimuli drive strong oscillations that are not observed during natural scenes.** A) Z-scored power spectra for LFP and B) MUA during 1.875 Hz grating stimulus (speed 1.5 degree/s and spatial frequency 1.25 cycles per degree) (green) and natural scenes movies (black). C) Frequency dependent coherence between LFP and MUA. Z-scored power spectra were determined by first calculating the multi-taper power spectra of spontaneous activity, activity during grating stimuli and during natural scenes stimuli. Then the spontaneous activity power in each frequency bin was subtracted from both the grating and natural scenes power and this was normalized by the spontaneous power’s standard deviation.
(TIF)

Figure S9 **“Sharp” LFP oscillations cause crosstalk between frequencies.** A) sMRA of a 70 Hz sawtooth (black) involves high frequency harmonics (colored curves) to capture its “sharpness”. B) “Preferred” LFP scale phases (at which the GLM predicts the highest probability of spiking) of the 44, 89 and 178 Hz scales compared across all neurons. C) Scatterplot of preferred phases reveals a strong correlation between the scales, indicating that the scales represent different aspects of the same underlying oscillation.
(TIF)

Table S1 **Percentages of neurons for which different nested models passed statistical validation tests.** LL: The log likelihood of the test data was greater for the more complicated model than for the next simplest model. AIC: The Akaike Information Criterion of the more complex model was smaller than that of the simpler model. BIC test: Same but using the Bayesian Information Criterion. KS test: Kolmogorov Smirnov time rescaling test was passed on the test data.
(TIF)
