# Supplementary material for: Context Matters: The Illusive Simplicity of Macaque V1 Receptive Fields
Source: PLoS One. 2012 Jul 3;7(7):e39699. doi: 10.1371/journal.pone.0039699 (PMC3389039; doi:10.1371/journal.pone.0039699)
Supplement: Text S1 — Details on spike sorting, control of LFP “leakage” into spikes, calculation of confidence bounds on GLM fits, goodness of fit of GLM to trial averaged PSTHs and the effect of eccentricity on results. (DOC) [file pone.0039699.s001.doc]

**Supplementary Text S1**

**Haslinger, Pipa, Lima, Singer, Brown and Neuenschwander**

**Supplementary Methods and Results**

**Aperture Masks**

Supplementary Figure S1 shows three examples of aperture mask placement. Each mask was individually located and sized based upon the CRF of an electrode’s multi-unit activity. See methods for details. Supplementary Figure S2 shows PSTH difference measures for a subset of neurons for which the aperture size was varied. Results are relatively stable within the 30-70 pixel range.

**Spike Sorting**

Unit isolation was carried out by using an offline spike-sorting program developed by Nan-Hui Chen at the Max-Planck Institute for Brain Research. This program uses a semiautomatic clustering procedure based on template-matching (residual sum of squares). First, the distances between the spikes and the templates were computed and, second, a similarity index threshold was calculated to decide how many units were present and if a spike should be assigned to a particular template. Sorting was initiated by leaving the template-matching algorithm to find as many clusters as possible automatically. We then switched to a manual mode in which the experimenter could decide which clusters to merge, if any. The program provided graphical and analytical tools, such as the refractory period seen in the autocorrelogram, to guide the merging decision. In addition, a change in waveform amplitude over time was computed for each cluster to identify and reject any single units showing significant instability. Correct spike assignment was further validated if distinct clusters could be seen in three-dimensional plots of spike principal component analysis scores, and if the overall separation of these clusters could be confirmed by objective measurements provided by the J3 and Pseudo-F statistics (details on how these values are calculated can be found in [1-2]. See Supplementary Figure S3 for spike waveforms of the three neurons who’s PSTHs are presented in this paper (Figure 1 and Supplementary Figure S5).

**Spike-LFP Leakage Control**

Since we compared spikes and LFPs recorded from the same electrode, it was crucial to determine the extent to which the spikes “leaked” into the LFP and whether such leakage influenced the GLM fits significantly. To determine the extent to which the spikes had been filtered out of our LFPs we used the LFP and the spike waveforms to generate two “compound LFPs”. That is, we added spike waveforms to the LFP (at the original (experimental) spike times for the first compound, and at altered spike times for the second) and then re-filtered the spikes out, using the exact same recording equipment that was used to collect the original data. The aim was to retain the relation between spikes and LFP in the first case, but destroy it in the second, while retaining leakage artifacts for both.

In detail, the compound signals were constructed by adding the original LFP (acquired at 1kHz) and spiking waveforms (acquired at 32 kHz) after up-sampling the two signals to 64 kHz In the first compound signal, the original timing of the spikes was used for placing the spike waveforms. In the second compound signal the timing of the spikes was altered by temporally reversing them. This destroyed the original correspondence of the spike timing and LFP. After the compound signals were reconstructed they were used to generate analog signals at 64 kHz (NI boards DAC), attenuated by a factor of 10000X and fed to the data acquisition system, replicating exactly the signal amplitude and conditioning during the experiments. This was done for 44 neurons, chosen as those for which inclusion of the LFP in the GLM of the original data produced the largest improvement in validation log likelihood. That is we used the neurons for which our methods deemed the LFP most important.

We then performed two controls using these compound signals. In the first we calculated spike triggered averages (STAs) of the 1) the original (not compound) LFP triggered upon the original spike times, 2) the LFP from the first compound signal (generated using the original spike times) triggered upon the original spike times 3) the LFP from the second compound signal (generated using the randomized spike times) triggered upon the altered spike times. In Supplementary Figure S4 A) we show these STAs for 9 characteristic neurons. The STAs generated from the first compound signal (triggered upon the original spike times) is highly similar to the STA generated from the original LFP. This demonstrates that our procedure for generating compound LFPs is valid. The STAs generated from the second compound signal (triggered upon the altered spike times) show minimal features when compared to the other two STAs. This demonstrates that the leak, while present, is much smaller than the LFP features, which are correlated with the spikes.

As a second control, we compared the importance of the LFP versus the leak on a neuron by neuron basis by fitting GLM models to the two control data sets. We then compared the increase in log likelihood (effectively the increased goodness of fit) when the LFP was included in the models. This allows us to quantify, in a statistically rigorous manner, the influence of the leak upon our GLM fits. In Supplementary Figure S4 B we show a histogram (over all 44 neurons) of the training data likelihood increase (when the LFP is included) of the altered time control data, normalized by the likelihood increase of the original time control data. This ratio was less than 0.2 for 70% of the neurons used. The fact that the LFP did improve the fit of some randomized control neurons indicates the presence of some leak. However, the influence of the leak on the log likelihood was, on average, one fifth that of the non-leak LFP. Further, in Supplementary Figure S4 C we show a scatter plot of the log likelihood increases for the original versus the control data. The control log likelihood has a range one quarter that of the original data and the scatter plot is essentially flat. Thus although a leak is present, its influence is minimal compared to that of the non-leak LFP.

**Confidence bounds on GLM fitted PSTHs**

Model 3 produced three different time varying, stimulus locked, firing rates (PSTHs) for the FF, AM and TR movies. To calculate the percentage of time two PSTHs were statistically (at the 95% confidence level) we used error propagation to calculate 95 % confidence bands upon the differences between the two PSTHs. Logistic regression models have of the form

$$\log\left[\frac{\lambda_{t}}{1-\lambda_{t}}\right]=\Lambda_{t}=X_{t}^{T}\theta$$

In the above $X_{t}$is a time dependent vector of covariates, $\theta$is a time independent vector of parameters fit by maximum likelihood estimation (MLE) and $\Lambda_{t}$ is convenient shorthand. Parameters fit by MLE is that parameters are asymptotically normally distributed about the maximum likelihood estimate $\theta^{MLE}$and the covariance matrix of the parameters $C(\theta)$can be obtained. The diagonal of this matrix is, respectively, the variance of each fitted parameter. Via standard propagation of error methods we note that $\Lambda_{t}=X_{t}^{T}\theta$ is normally distributed about the maximum likelihood estimate $\Lambda_{t}^{MLE}=\log\left[\frac{\lambda_{t}^{MLE}}{1-\lambda_{t}^{MLE}}\right]$with variance $\sigma_{t}^{2}=X_{t}^{T}CX_{t}$.

Confidence bands for the time varying firing probability can be obtained by calculating the cumulative density function (CDF), making a variable transform and inverting. The CDF is given by

$\Phi(x;\mu,\sigma^{2})$is the Normal CDF with mean $\mu$ and variance$\sigma^{2}$. The above equation is easily inverted to give the quantiles of $\lambda_{t}$.

$$\lambda_{t}(p)=\frac{\exp\Lambda_{t}(p)}{1-\exp\Lambda_{t}(p)}$$

where

$$\Lambda_{t}(p)=\log\frac{\lambda_{t}}{1-\lambda_{t}}+\sqrt{2}\sigma_{t}erf^{-1}(2p-1)$$

95% confidence bounds on $\lambda_{t}$are obtained by setting p=0.025 and 0.975.

The PSTH is a sum of spikes over repeated trials and is a nonlinear function of $\Lambda_{t}$. To obtain confidence bands upon the PSTH and the difference between PSTHs we resort to approximate methods. Any nonlinear function $f(\Lambda)$ of $\Lambda$ will be approximately normally distributed with variance $(\partial f(\Lambda_{t})/\partial\Lambda_{t})^{2}\sigma_{t}^{2}$, where this result follows from expanding $f(\Lambda)$ in a Taylor series. To get the PSTH expected from the logistic regression model, a sum of spike probabilities over repeated stimulus trials is required.

$$PSTH_{t}^{MLE}=\frac{1}{N}\sum_{i=1}^{N} \lambda_{t,i}^{MLE}=\frac{1}{N}\sum_{i=1}^{N} \frac{\exp[\Lambda_{t,i}^{MLE}]}{1+\exp[\Lambda_{t,j}^{MLE}]}$$

The approximate variance of this function is

$$\sigma_{PSTH,t}^{2}=\frac{1}{N}\sum_{i=1}^{N} \left[\lambda_{t,i}^{MLE}(1-\lambda_{t,i}^{MLE})\right]^{2}\sigma_{t,i}^{2}$$

Thus approximate 95% confidence bands upon the PSTH can be obtained from the p=0.025 and 0.975 quantiles of the Normal distribution with mean $PSTH_{t}$ and variance $\sigma_{PSTH,t}^{2}$.

$$PSTH_{t}(p)=PSTH_{t}^{MLE}+\sqrt{2}\sigma_{PSTH,t}erf^{-1}(2p-1)$$

Confidence bands upon the *difference* between two PSTHs can be obtained as in a similar manner using the mean

$$\Delta PSTH_{t}=PSTH_{1}-PSTH_{2}$$

and the variance

$$\sigma_{\Delta PSTH,t}^{2}=\sigma_{PSTH_{1},t}^{2}+\sigma_{PSTH_{2},t}^{2}$$

It should be noted that since the error on the fitted parameters is normally distributed, the width of the confidence bounds scales roughly as $1/\sqrt{(N)}$where N is the number of trials used to calculate the PSTH.

**Additional Example Neurons**

PSTHs and model derived error bars are shown for two additional neurons in Supplementary Figure S5. Color scheme is analogous to that of Figure 1 in the main text.

**Non-parametric Basis Spline Fits**

Splines were defined by knots spaced 20 ms apart chosen to match the movie frame rate of 50 Hz. We were interested in how faithfully the GLM fits matched the raw PSTH (calculated in 20 ms bins). We show two example training data PSTHs and their spline fits in Supplementary Figure S6 A. Across the entire data set, 90% of neurons had correlation coefficients between the GLM and training data PSTH greater than r=0.9 (Figure S6 B left). Neurons with low correlation coefficients tended to have very low ~1Hz firing rates. The correlation coefficient, corrected for finite data size is the *explained variance* of David and Gallant [3]. We calculated this for the test data both with and without correcting for the finite number of test data trials (see [3] for details on finite size corrections). As shown in Figure S6 B (middle and right), 70% (population median) of the explained variance could be accounted for prior to correcting for finite test data size, and 91% after correction. For comparison, David and Gallant report 40% explained variance accounted for by their forward model after correcting for finite data sizes [3].

**Effect of Eccentricity**

There was no correlation between eccentricity and aperture size (r=-0.02; pval=0.77 Pierson's Correlation Coefficient). However there could be other reasons that the surround would have a different influence for cells with CRFs at 2-5 degrees than it does for cells located at 10-14 degrees. In Supplementary Figure S7 we investigated how our three population measures of Figure 1 (percentage of PSTH which was different between FF and AM (or TR), the normalized difference between the PSTHs, and the normalized difference in the mean firing rates) changed as a function of eccentricity. That is, for each measure, we determined the distributions for cells from 2-5 degrees and cells from 10-14 degrees separately and compared these distributions using non-parametric Kolmogorov Smirnov tests. All distribution pairs (except one) were statistically identical (p>0.05 via KS test). The distributions for normalized mean firing rate change between FF and TR movies were barely significantly different (p=0.049) however the medians of these two distributions were statistically identical via non-parametric rank sum test (p=0.07).

**LFP and MUA Power Spectra and Coherence**

The power spectra for both LFP and MUA change drastically between grating and natural scenes stimuli, as does the coherence between the two. This suggests that gratings induce an artificial rhythmicity to the V1 network dynamics. See Supplementary Figure S8.

**Supplementary References**

1. Spath, H. (1980) Cluster Analysis Algorithms for Reduction and Classification of Objects. Ellis Horwood, West Sussex, UK.

2. Wheeler, B.C. (1999) Automatic discrimination of single units. In Nicolelis, M.A.L. (Ed.), Methods of Neural Ensemble Recording. Methods in Life Sciences. CRC Press, Boca Raton, FL, pp. 61–78.

3. David S.V. and Gallant J.L. (2005) Predicting neuronal responses during natural vision. Network 16: 239-260.
