# Supplementary figures and images for: Context Matters: The Illusive Simplicity of Macaque V1 Receptive Fields
Source: PLoS One. 2012 Jul 3;7(7):e39699. doi: 10.1371/journal.pone.0039699 (PMC3389039; doi:10.1371/journal.pone.0039699)

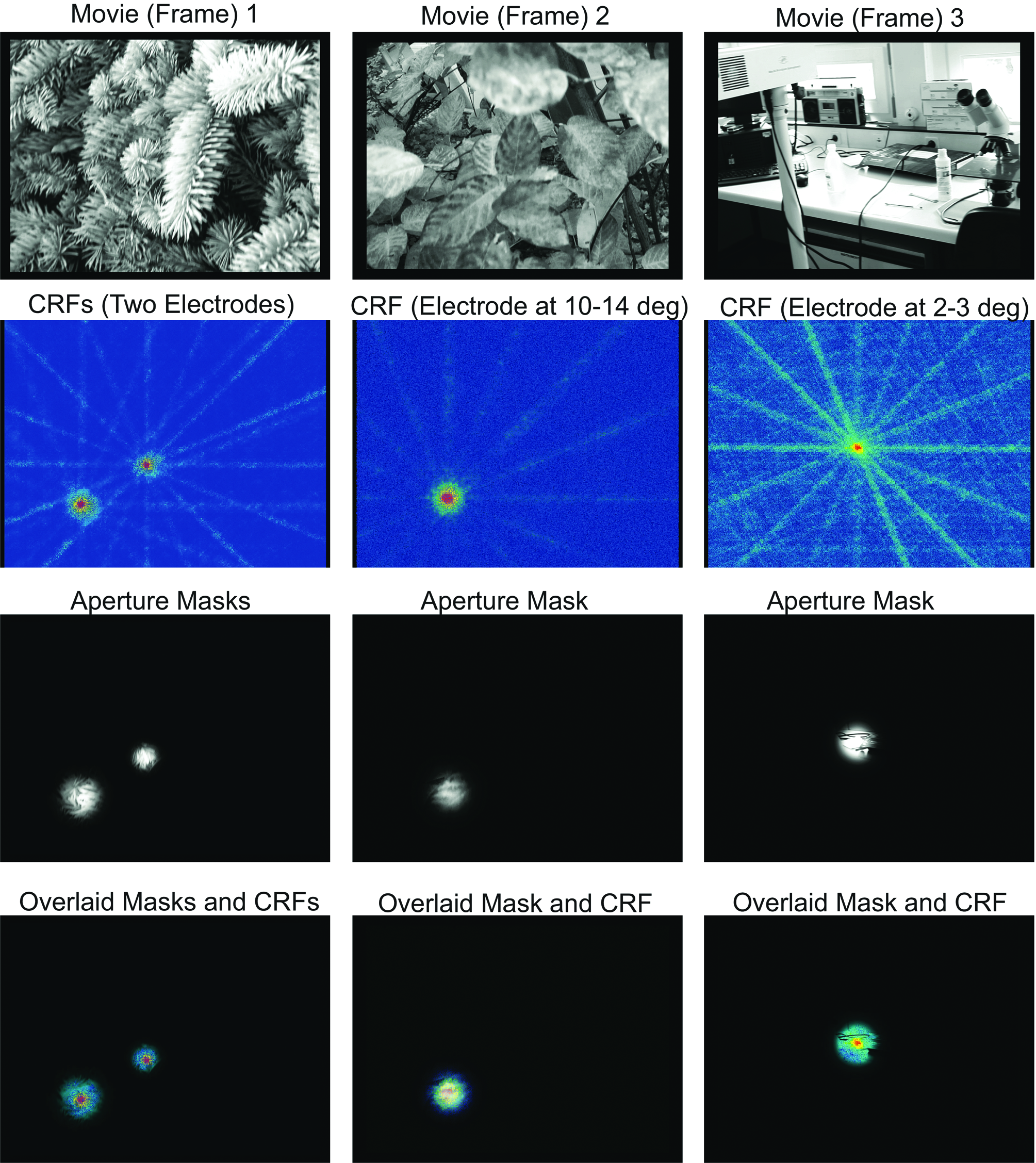

Supplement: Figure S1 — Three examples (columns) of aperture mask placement. Top row: movie frame. Second row: CRFs of multiunit activity of recording electrodes. (Example in left column records from both 2-5 degrees eccentricity and 10-14 degrees, i.e. two different electrodes). Third row: aperture masks generated on-line. Bottom row: aperture masks overlaid on CRFs. Note that masks fully contain CRFs. (TIF) [file pone.0039699.s002.tif]

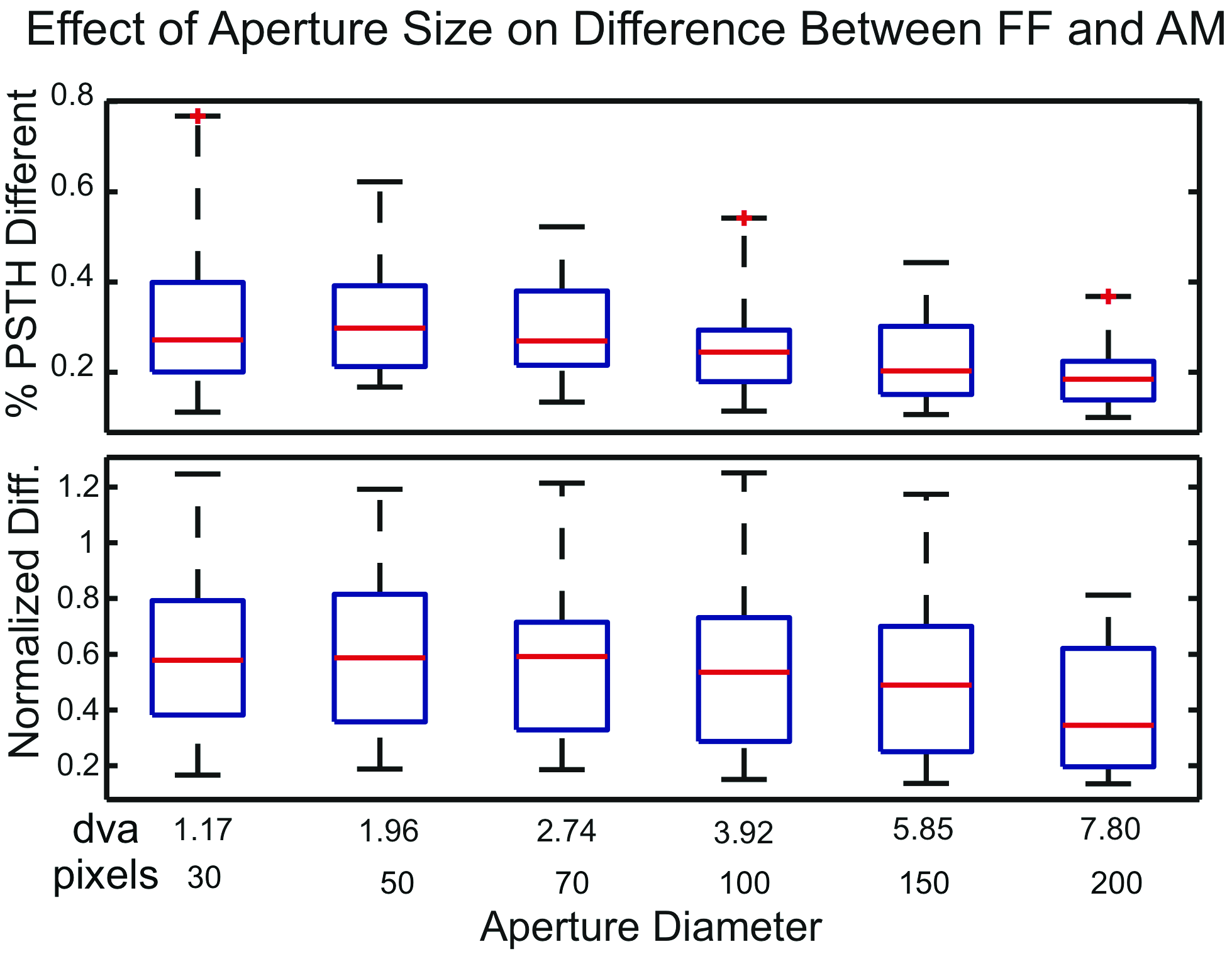

Supplement: Figure S2 — Varying aperture mask size. The percentage of the PSTH that was statistically different (at 95% confidence levels) between FF and AM movies (upper panel) and the normalized difference in time varying firing rates (lower panel) for different sized apertures. There are (38, 13 38, 38, 38, 19) neurons for the (30, 50, 70, 100, 150, 200 pixel) diameter apertures respectively. Corresponding diameters in degrees of visual angle are given in the figure. (TIF) [file pone.0039699.s003.tif]

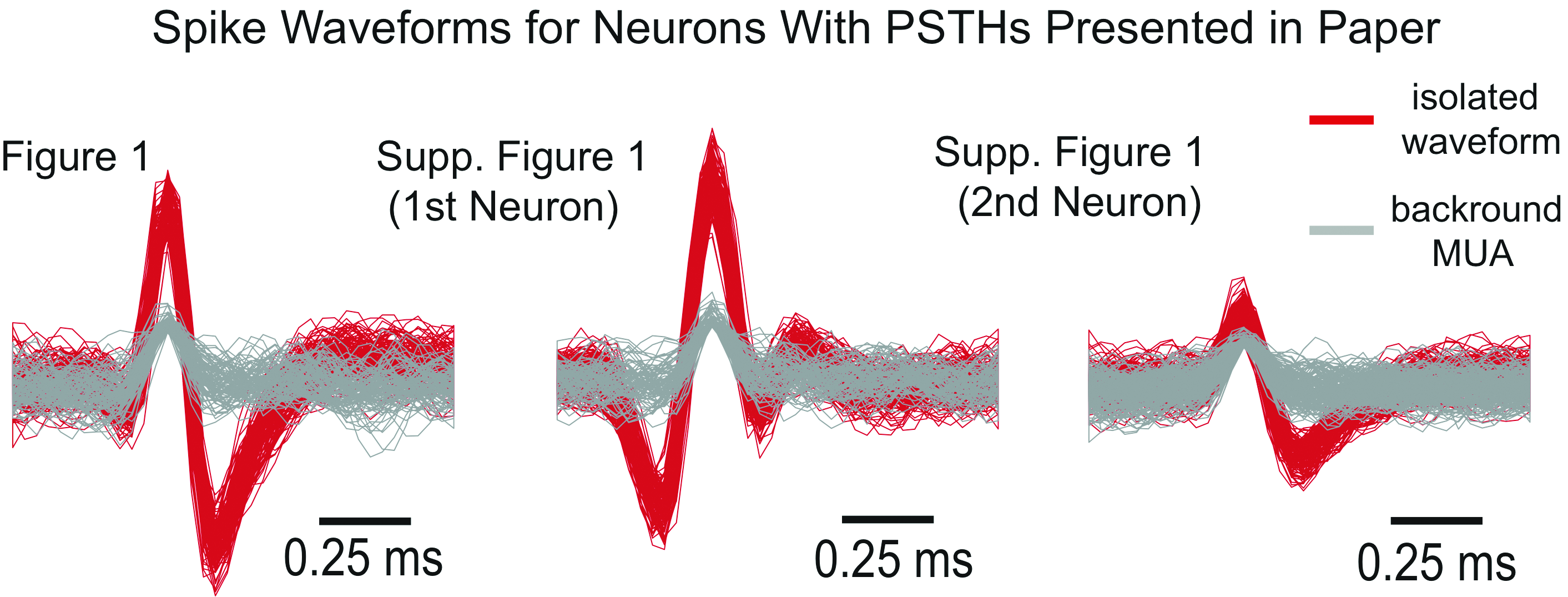

Supplement: Figure S3 — Spike waveforms isolated from multiunit activity. Waveforms of the three neurons whose PSTHs are presented in the paper (Figure 1 & Figure S4) are shown in red. Grey shows non-isolated background spikes (MUA). (TIF) [file pone.0039699.s004.tif]

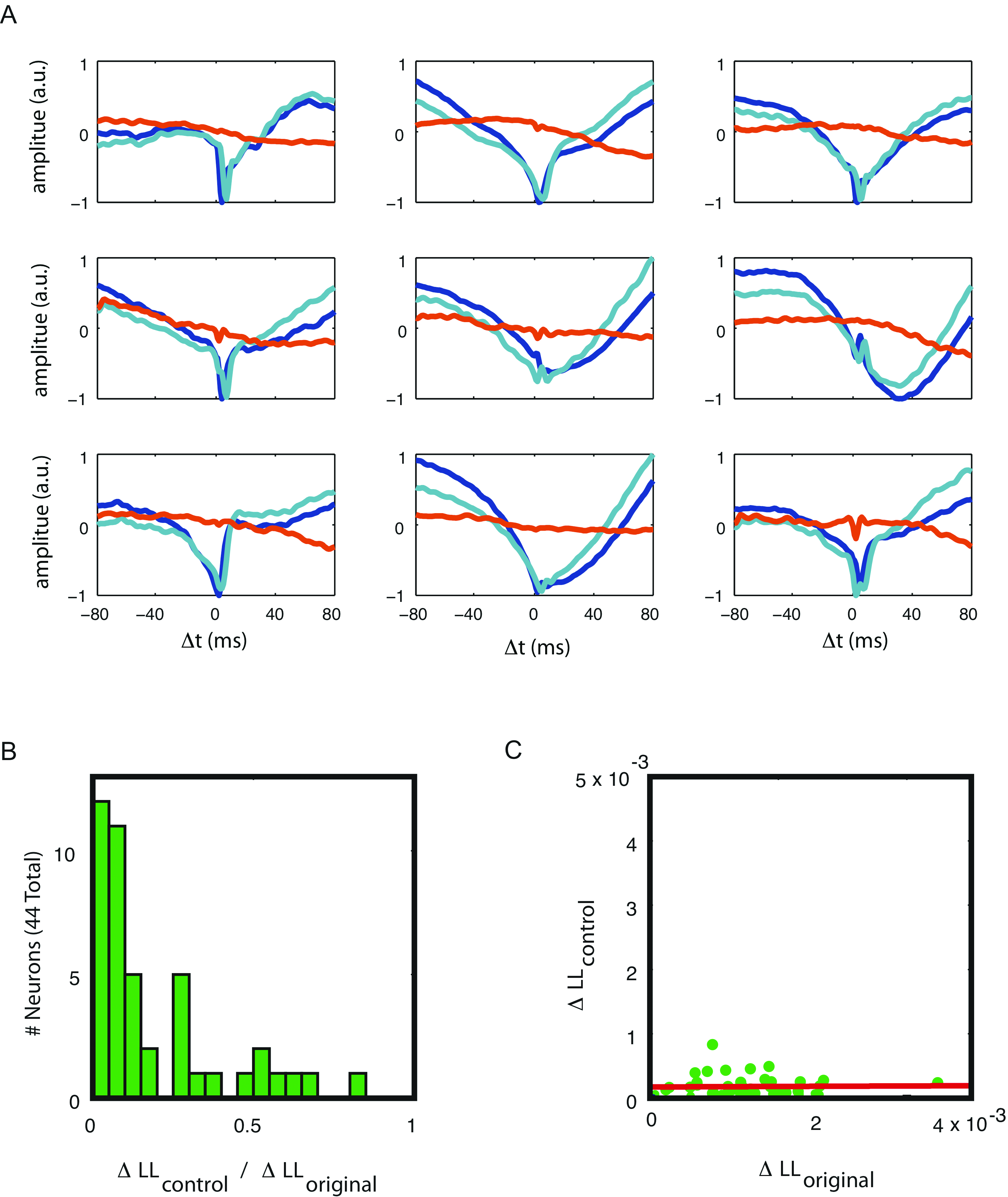

Supplement: Figure S4 — Quantifying spike leakage into the LFP. A) Spike triggered averages from 9 representative neurons. Dark blue: STA of original LFP, Light blue: STA of compound LFP generated using original spike times, Red: STA of compound LFP generated using altered spike times. STAs of the original and first compound LFPs are highly similar indicating that our procedure for generating compound LFPs works properly. The STA of the second (red) compound LFP is, in contrast negligible with only minor leakage effects. B) Histogram of log likelihood increase, upon inclusion of LFP in a GLM model, of the second (altered spike time) compound data normalized by the log likelihood of the first (original spike time) compound data. For 70% (out of 44 neurons shown) the control (altered spike time) data has a log likelihood increase less than one fifth (20%) that of the original data. C) Scatter plot of the LFP induced increase in log likelihood for the original and control data. Each dot represents a single neuron. Red line is a linear regression. (TIF) [file pone.0039699.s005.tif]

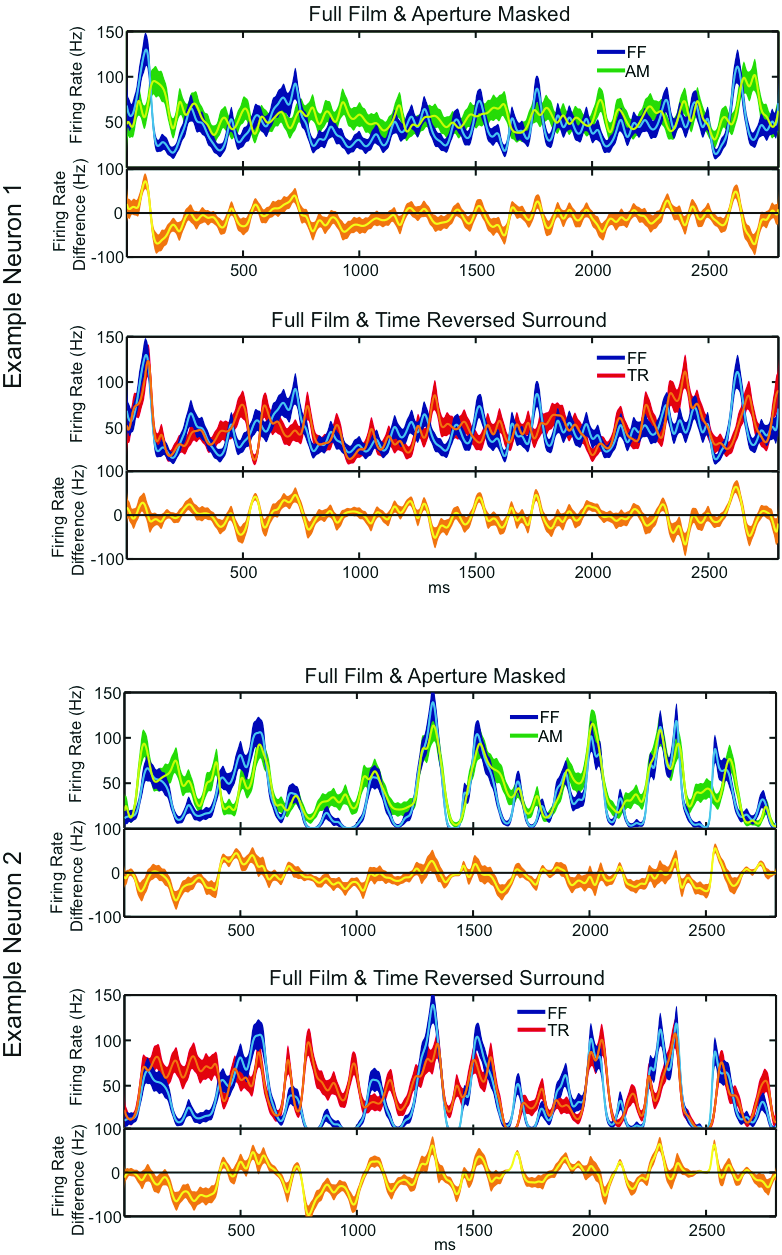

Supplement: Figure S5 — PSTHs of two additional V1 neurons. These exhibit significantly different stimulus locked firing responses to natural scenes stimuli when the surround is changed but the CRF stimulus is not. As in the main text, upper panels show GLM fitted “PSTHs” (blue = FF, green = AM, red = TR) and lower panels show differences (in yellow) between PSTHs. Lighter lines are the PSTHs and differences while the dark bands denote 95% confidence regions. (TIF) [file pone.0039699.s006.tif]

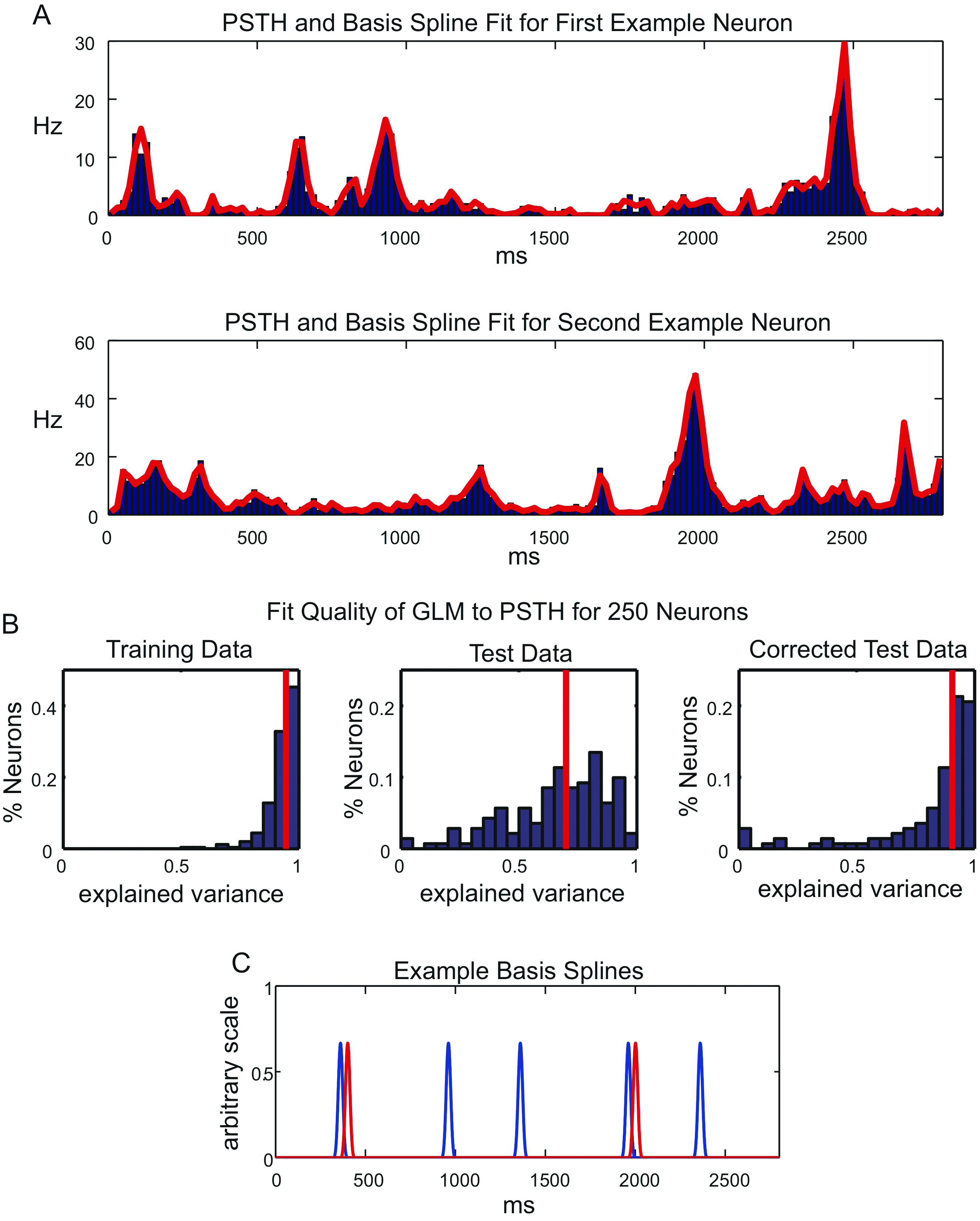

Supplement: Figure S6 — Spline based GLM models accurately fit trial averaged firing rate (PSTH). A) PSTHs (20 ms histogram) and spline fits (red) for two example neurons under natural scenes stimulation. B) Distribution of explained variance of training data (left), test data (middle) and test data corrected for finite number of test data trials (right). C) Splines used to non-parametrically model the stimulus drive tiled the entire 2800 ms span. Here we show a subset. Colors are visual aid to distinguish adjacent splines. (TIF) [file pone.0039699.s007.tif]

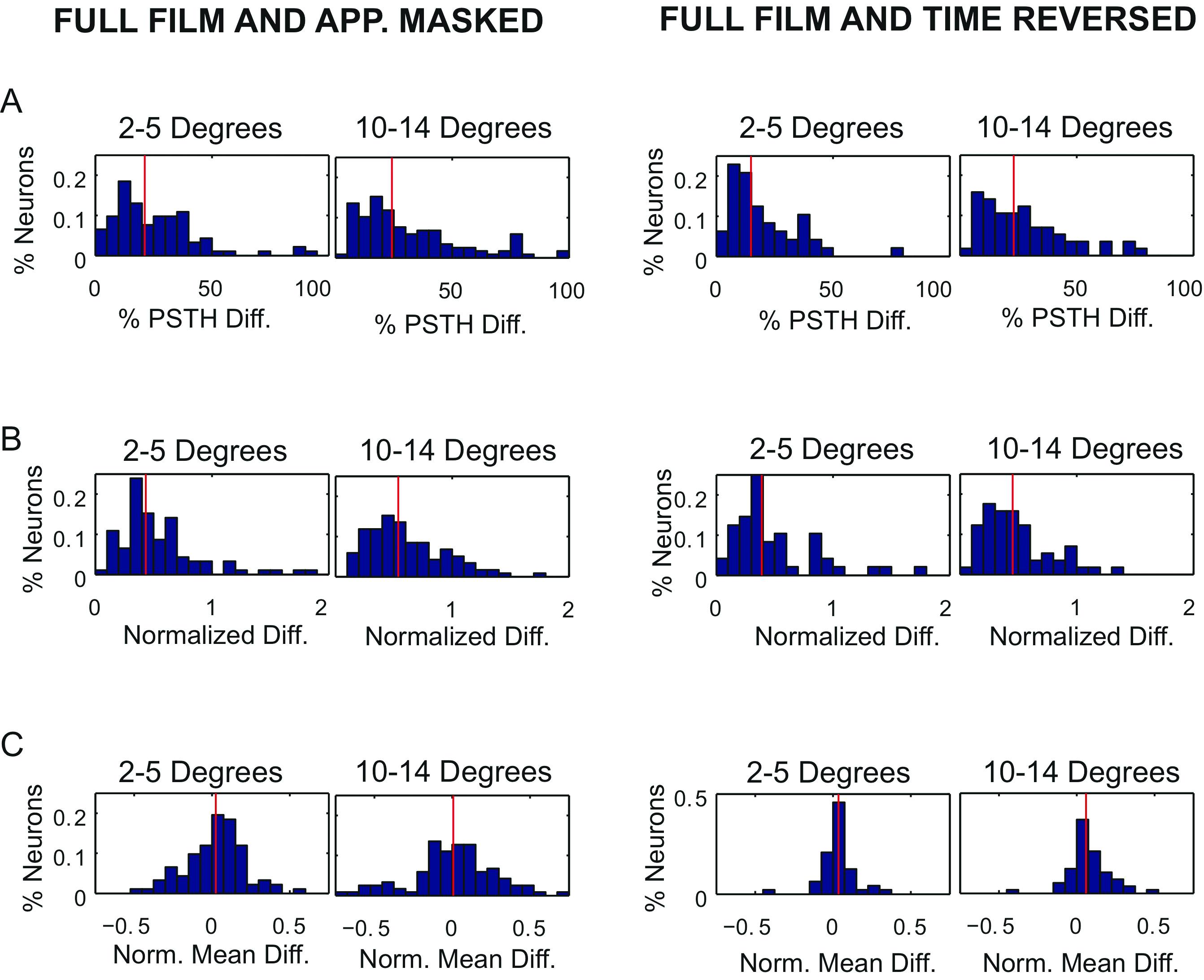

Supplement: Figure S7 — Comparing differences between the PSTHs as a function of eccentricity (2-5 degrees versus 10-14 degrees). A) Percentage of PSTH statistically different, B) normalized difference between PSTHs, C) normalized mean firing rate difference between PSTHs. Distributions are all identical (via KS test) between 2-5 and 10-14 degrees except for the normalized mean firing rate difference between FF and TR (p = 0.049). (TIF) [file pone.0039699.s008.tif]

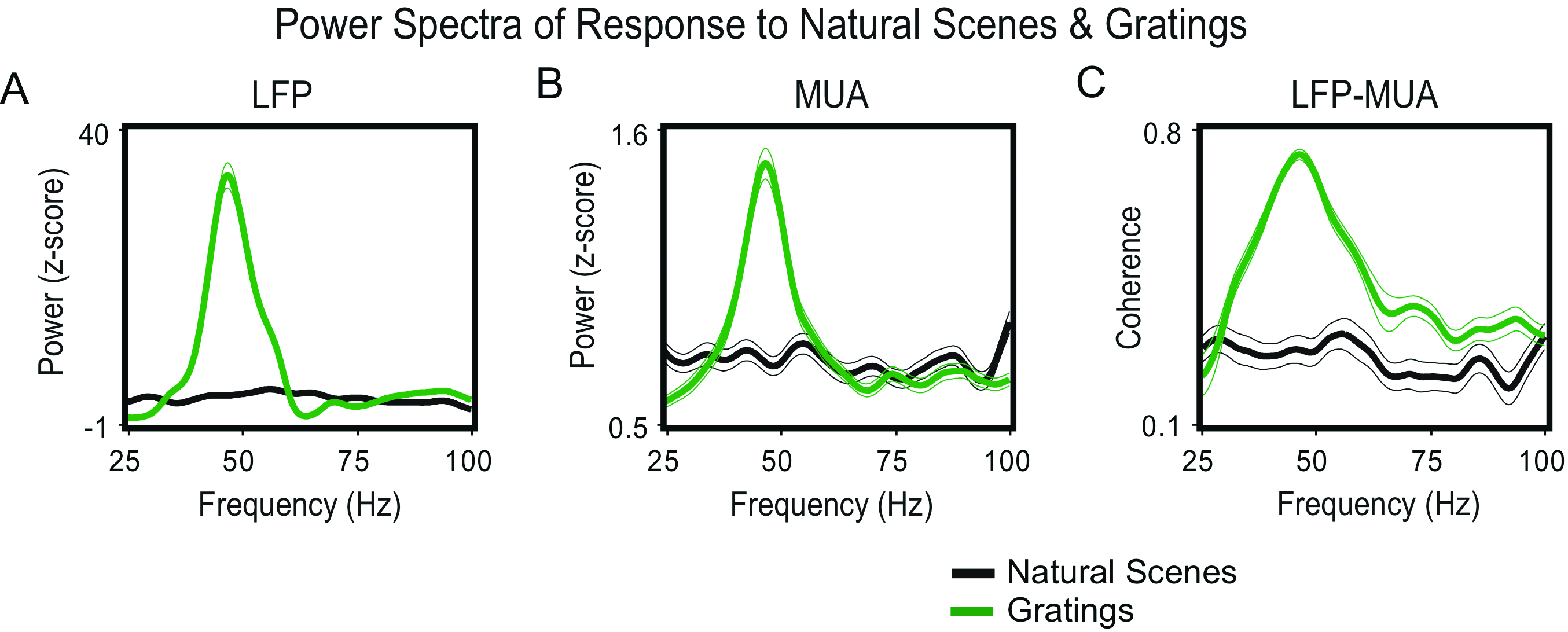

Supplement: Figure S8 — Grating stimuli drive strong oscillations that are not observed during natural scenes. A) Z-scored power spectra for LFP and B) MUA during 1.875 Hz grating stimulus (speed 1.5 degree/s and spatial frequency 1.25 cycles per degree) (green) and natural scenes movies (black). C) Frequency dependent coherence between LFP and MUA. Z-scored power spectra were determined by first calculating the multi-taper power spectra of spontaneous activity, activity during grating stimuli and during natural scenes stimuli. Then the spontaneous activity power in each frequency bin was subtracted from both the grating and natural scenes power and this was normalized by the spontaneous power’s standard deviation. (TIF) [file pone.0039699.s009.tif]

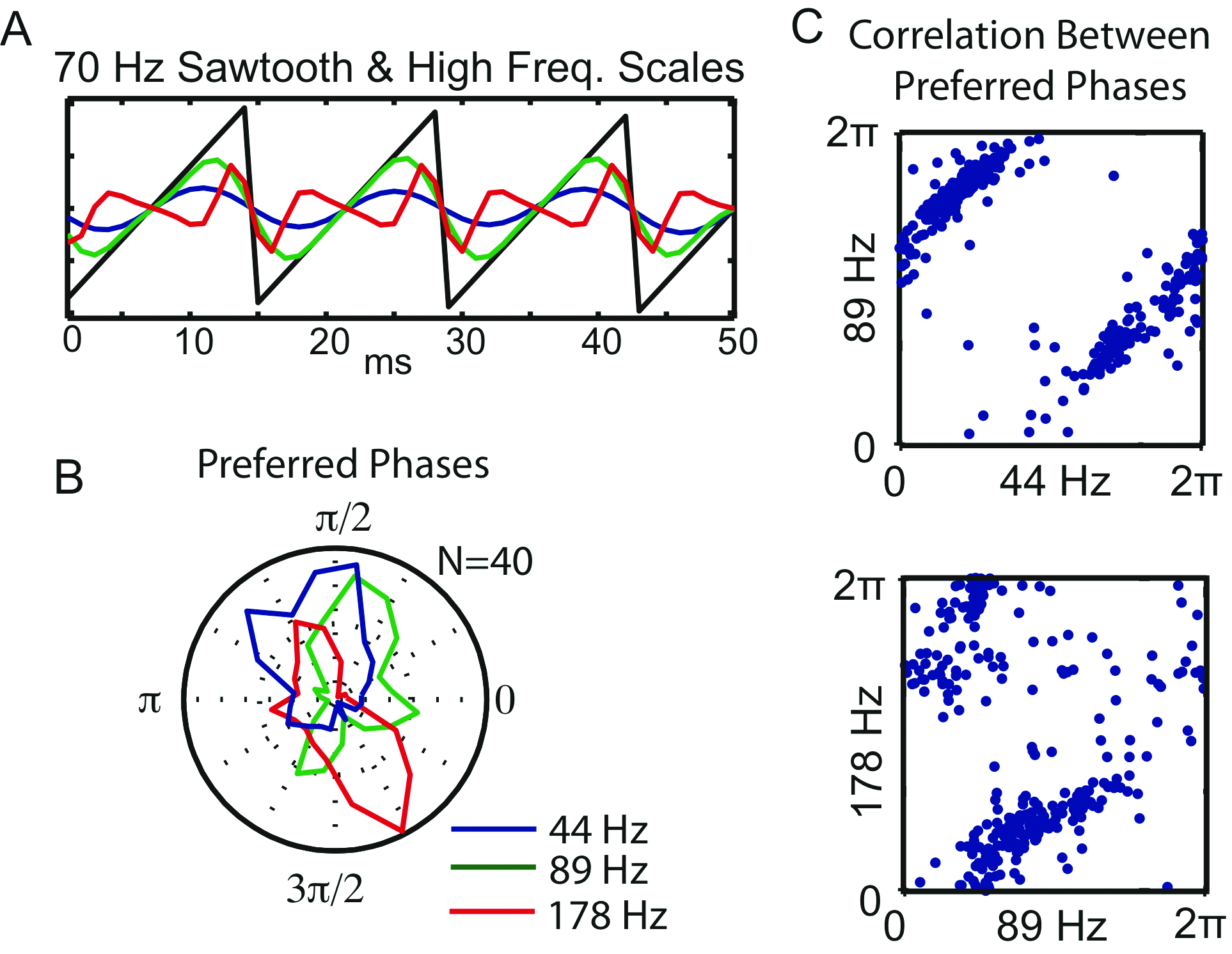

Supplement: Figure S9 — “Sharp” LFP oscillations cause crosstalk between frequencies. A) sMRA of a 70 Hz sawtooth (black) involves high frequency harmonics (colored curves) to capture its “sharpness”. B) “Preferred” LFP scale phases (at which the GLM predicts the highest probability of spiking) of the 44, 89 and 178 Hz scales compared across all neurons. C) Scatterplot of preferred phases reveals a strong correlation between the scales, indicating that the scales represent different aspects of the same underlying oscillation. (TIF) [file pone.0039699.s010.tif]

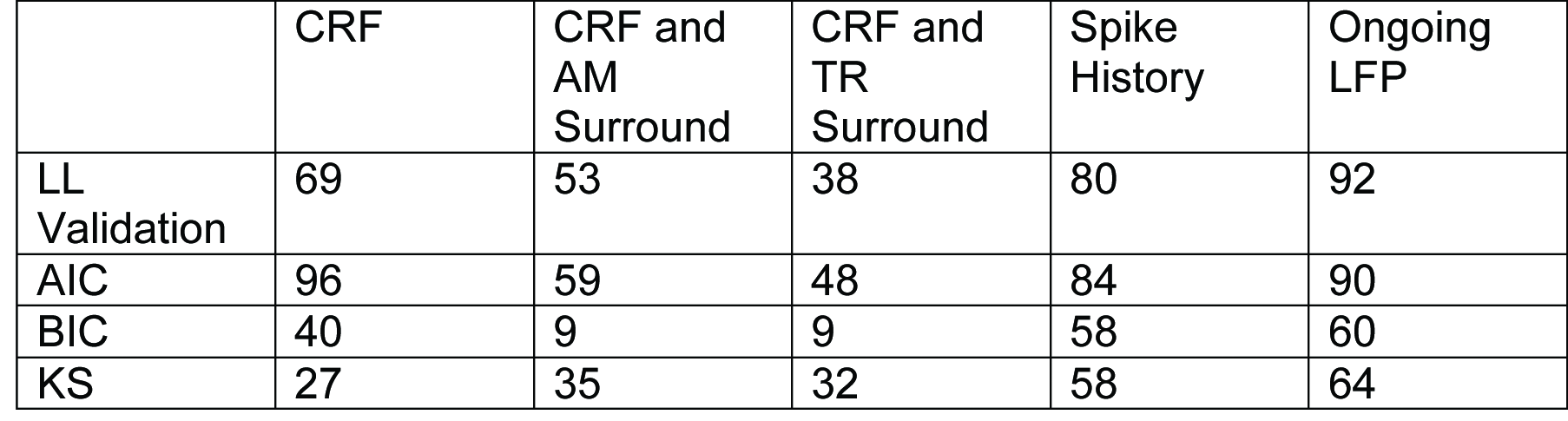

Supplement: Table S1 — Percentages of neurons for which different nested models passed statistical validation tests. LL: The log likelihood of the test data was greater for the more complicated model than for the next simplest model. AIC: The Akaike Information Criterion of the more complex model was smaller than that of the simpler model. BIC test: Same but using the Bayesian Information Criterion. KS test: Kolmogorov Smirnov time rescaling test was passed on the test data. (TIF) [file pone.0039699.s011.tif]
